# Wuji Pill and *Akkermansia muciniphila* alleviates intestinal dysfunction and depression-like behavior in irritable bowel syndrome through the microbiota-gut-brain axis

**DOI:** 10.3389/fmicb.2026.1739408

**Published:** 2026-02-04

**Authors:** Mengting Li, Shuiming Xiao, Yanli Wang, Tao Li, Qin Hu, Lijinchuan Dong, Yuxuan Guo, Zhe Shi, Qing Yang, Weiyan Cai, Qi Li, Bo Peng, Pengyue Li, Xiaogang Weng, Yajie Wang, Yujie Li, Yu Dong, Xiaoxin Zhu, Zipeng Gong, Ying Chen

**Affiliations:** 1 Institute of Chinese Materia Medica, China Academy of Chinese Medical Sciences, Beijing, China; 2 State Key Laboratory of Discovery and Utilization of Functional Components in Traditional Chinese Medicine, Guizhou Medical University, Guiyang, China; 3 Experimental Research Center, China Academy of Chinese Medical Sciences, Beijing, China; 4 College of Chemistry and Life Science, Beijing University of Technology, Beijing, China; 5 Division of Stem Cell Regulation and Application, Key Laboratory for Quality Evaluation of Bulk Herbs of Hunan Province, Hunan University of Chinese Medicine, Changsha, China; 6 Guang'anmen Hospital, China Academy of Chinese Medical Sciences, Beijing, China

**Keywords:** *Akkermansia muciniphila*, irritable bowel syndrome, microbiota-gut-brain axis, tryptophan metabolism, Wuji Pill

## Abstract

**Introduction:**

Irritable bowel syndrome (IBS) is a typical disorder of gut-brain interaction (DGBI). The microbiota-gut-brain (MGB) axis is pivotal in preventing and treating IBS. Wuji Pill is a traditional Chinese medicine commonly used to treat IBS. This study aimed to investigate the mechanism by which Wuji Pill improves IBS via the MGB axis.

**Methods:**

The visceral sensitivity and colonic motor function were evaluated using the abdominal wall withdrawal reflex test and the colonic motility curve. Depression-like behavior were evaluated using sucrose preference test, open field test, novelty-suppressed feeding test, and forced swimming tests. The intestinal mucus secretion and the activation status of microglia was detected using AB-PAS staining and immunofluorescence staining, respectively. The species composition and abundance of gut microbiota were detected through 16S rRNA sequencing and RT-qPCR. Targeted metabonomics and RT-qPCR were used for metabolites and metabolic enzymes analysis.

**Results:**

In this study, Wuji Pill improved the symptoms of IBS rats and increased the relative abundance of *Akkermansia muciniphila* in feces. Additionally, antibiotics affected the repair of intestinal mucus secretion and significantly reduced the level of short-chain fatty acids. Subsequently, fecal microbiota transplantation and *A. muciniphila* transplantation can improve the symptoms of IBS rat by increasing intestinal mucus secretion, elevating the levels of acetic acid and butyric acid in feces. Additionally, the microglia in the cortex were suppressed, and the tryptophan-kynurenine pathway in the hippocampus was inhibited, leading to the conversion of tryptophan into 5-HT.

**Discussion:**

This study highlights the Wuji Pill may alleviate IBS symptoms by modulating *A. muciniphila* and regulating the tryptophan metabolism pathway through MGB axis.

## Graphical abstract

**Figure:**
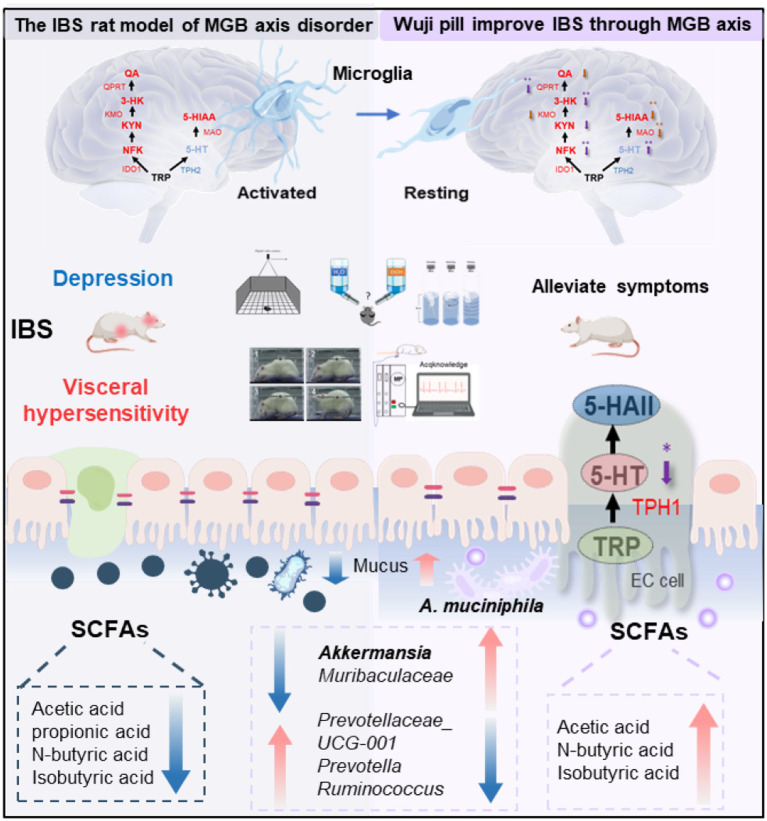

## 1 Introduction

Irritable bowel syndrome (IBS) is a classic disorder of gut-brain interaction (DGBI) characterized by abdominal pain, bloating, and changes in bowel habits (Drossman and Hasler, 2016). According to the Meta-Analysis, the overall prevalence of IBS was 14% (Arif et al., 2024). As a psychosomatic disease, it has been reported that approximately one-third of IBS patients exhibit symptoms of anxiety and depression (Staudacher et al., 2023), which are commonly observed in adult women. Clinical guidelines recommend the management of IBS from both diagnostic and treatment aspects (Lacy et al., 2021). However, the pathogenesis of IBS remains unknown. In the pathophysiological mechanism, IBS may be associated with visceral hypersensitivity, intestinal motility, intestinal epithelial barrier, gut microbiota, brain-gut axis, immune regulation, food antigens, and bile acid metabolism (Ford et al., 2020). Therefore, it is particularly important to elucidate the pathophysiological mechanisms of IBS and explore its diagnostic and therapeutic strategies.

Dysfunction of the microbiota-gut-brain (MGB) axis is considered one of the important pathophysiological mechanisms for IBS accompanied by neurological diseases. Disruption of the “microbiota-gut-brain axis” may serve as the central hub linking intestinal symptoms to neurological disorders (Wu et al., 2022). IBS is characterized by gut microbiota dysbiosis, which leads to impaired barrier function and the production of abnormal metabolites. In addition to affecting the gastrointestinal symptoms of IBS, gut microbes also influence the nervous system (Hillestad et al., 2022). Gut microbiota and metabolites can directly or indirectly affect brain activity (Mishima and Ishihara, 2020), while gut microbiota is regulated by the central nervous system (Toledo et al., 2025). Tryptophan metabolism, as one of the ways of regulating the microbiota-gut-brain (MGB) axis, involves the interaction of a variety of enzymes and metabolites (Platten et al., 2019). Studies have shown that there was a correlation between tryptophan metabolism and IBS with comorbid depression, showing significantly elevated circulating levels of kynurenine, tryptamine, and histamine in depressed patients (Han et al., 2022). Under conditions of excessive activation of the tryptophan-kynurenine pathway, the deficiency of tryptophan and serotonin in the brain will lead to depressive symptoms. At present, pharmacological modulation of gut-brain axis dysfunction and targeted gut microbiota therapy (such as fecal microbiota transplantation, probiotics, prebiotics, etc.) are gradually used to treat IBS (Nee and Lembo, 2021; Weng et al., 2024), and the specific mechanism is still unclear (Yadegar et al., 2024).

As an effective strategy for improving psychosomatic diseases, Traditional Chinese Medicine (TCM) has the characteristics of holistic view in the treatment of diseases (Guo et al., 2023). TCM usually contains a variety of ingredients, which can achieve multi-target, multi-route and multi-link treatment (Li et al., 2024). For example, Berberine from Rhizoma Coptidis has been proved to play a pharmacological role through gut microbiota (Wang et al., 2021). Evodiamine and rutaecarpine in Fructus evodiae can exert analgesic effect (Holzer and Izzo, 2014). The active ingredients in Radix Paeoniae Alba have been demonstrated to improve the nervous system (Zhao et al., 2018). Wuji Pill is a TCM compound commonly used in the treatment of gastrointestinal diseases in China (Gong et al., 2022), which is composed of Rhizoma Coptidis, Fructus evodiae and Radix Paeoniae Alba. Preliminary studies have evaluated the pharmacokinetic and pharmacological mechanisms of Wuji Pill in improving IBS and post-inflammatory IBS in rats (Gong et al., 2014). However, the research on the improvement of nervous system by Wuji Pill has not been reported. Moreover, we found that Wuji Pill could reverse the decrease of gut microbiota abundance in post-inflammatory IBS rats and significantly increased the relative abundance of *Akkermansia*, *Bacteroides*, and *Parasutterella* (Chen et al., 2017). *Akermansia muciniphila* (*A. muciniphila*), a next-generation beneficial bacterium that has attracted considerable attention, can increase and play a role in repairing the intestinal barrier (Hughes et al., 2025). Furthermore, its function in modulating the gut-brain axis and maintaining host health is becoming increasingly prominent (Yuan et al., n.d.). A decrease in the abundance of *A. muciniphila* is associated with various gastrointestinal and mood disorders (Matijašić et al., 2025). Studies have shown that pasteurized *A. muciniphila* exerts analgesic and anti-anxiety effects by regulating TRPV1 and GPCRs to improve IBS (Meynier et al., 2024). After administration of Wuji Pill, the abundance of *A. muciniphila* increased significantly. However, the mechanism by which *A. muciniphila* improves IBS through the gut-brain axis remains unknown.

The potential mechanism by which Wuji Pill ameliorates IBS via the MGB axis has aroused our interest. Firstly, we established an IBS rat model with visceral hypersensitivity and depression. Moreover, we evaluated the effects of Wuji Pill alleviates intestinal dysfunction and depression-like behavior of IBS rats. In addition, a pseudo germ-free IBS rat model was used to validate the role of gut microbiota. Furthermore, we elucidated the mechanism of Wuji Pill in improving IBS through fecal microbiota transplantation (FMT) and *Akkermansia muciniphila* (*A. muciniphila*) transplantation experiments. This study confirmed for the first time that Wuji Pill ameliorates IBS through the MGB axis, which provides experimental evidence supporting therapeutic strategies for IBS.

## 2 Materials and methods

### 2.1 Reagents and materials

Acetic acid (H1808008), propionic acid (A1903124), n-butyric acid (E1814125), and isobutyric acid (K1822187) were purchased from Aladdin Biochemical Technology Co., Ltd. (Shanghai, China). Tryptophan (T0254), 5-hydroxytryptamine (H952), kynurenine (K8625), and 3-hydroxykynurenine (H1771) were purchased from Sigma. 5-hydroxyindoleacetic acid (121282500) was purchased from ACROS. Quinolinic acid (P63204) was purchased from ALDRICH.

*A. muciniphila* (ATCC BAA835) was purchased from BeNa Culture Collection (Beijing, China). BHI broth (CM917) was purchased from Beijing Land Bridge Technology Co., Ltd. (Beijing, China). Chen et al. in our research group had previously prepared Wuji Pills with stable quality, and Guo et al. had carried out sufficient qualitative and quantitative analyses (Chen et al., 2017; Guo et al., 2022). The chemical composition analysis of Wuji Pill from previous studies were provided in the Supplementary Figures S1, S2 and Supplementary Table S1.

### 2.2 Animals

Sprague–Dawley (SD) pregnant rats were provided by Beijing Weitonglihua Experimental Animal Technology Co., Ltd. (Certificate of Qualification: SCXK (Beijing, China) 2016-0011). All animals were housed fed with food and water ad libitum (temperature 22 ± 2 °C, 12 h light/dark cycle). All experiments were provided by the Institute of Chinese Materia Medica, Chinese Academy of Chinese Medical Sciences (SYXK (Beijing, China) 2020-0042, SYXK (Beijing, China) 2022B191). All animal experiments were strictly approved by the Animal Experiment Welfare and Ethics Committee of the Institute of Chinese Materia Medica, China Academy of Chinese Medical Sciences and followed the international guidelines for animal research.

### 2.3 Construction of irritable bowel syndrome model

The IBS combined with depression model was induced by exposure to multiple stressors during the juvenile and adult stages of female SD rats, as previously described (Gong et al., 2022). During the juvenile stage (from day 7 to day 21 of birth in rats), percutaneous transluminal coronary angioplasty (PTCA) balloon dilation catheter (specification: 3.0 mm × 20 mm, Cordis) stimulation, maternal separation (MS), and chronic restraint stress (CRS) were used for modeling. Firstly, a PTCA balloon (Paraffin lubricated) was inserted into the anus of neonatal rats for 2 cm, and the intestinal tract was stimulated for 1 min after the balloon was fully inflated. Secondly, after the balloon was removed, the forelimbs of neonatal rats were bound, and the MS stimulation was performed for 3 h (Varghese et al., 2006). Finally, the neonatal rats were stimulated again with PTCA balloon, and then put back into the cage. In the adult stage (from day 49 to day 70 of birth in rats), the rats were stimulated by CRS for 9 h every day (Reagan et al., 2004).

### 2.4 Treatments

#### 2.4.1 Drugs and Wuji Pill administration

**Figure 1:**
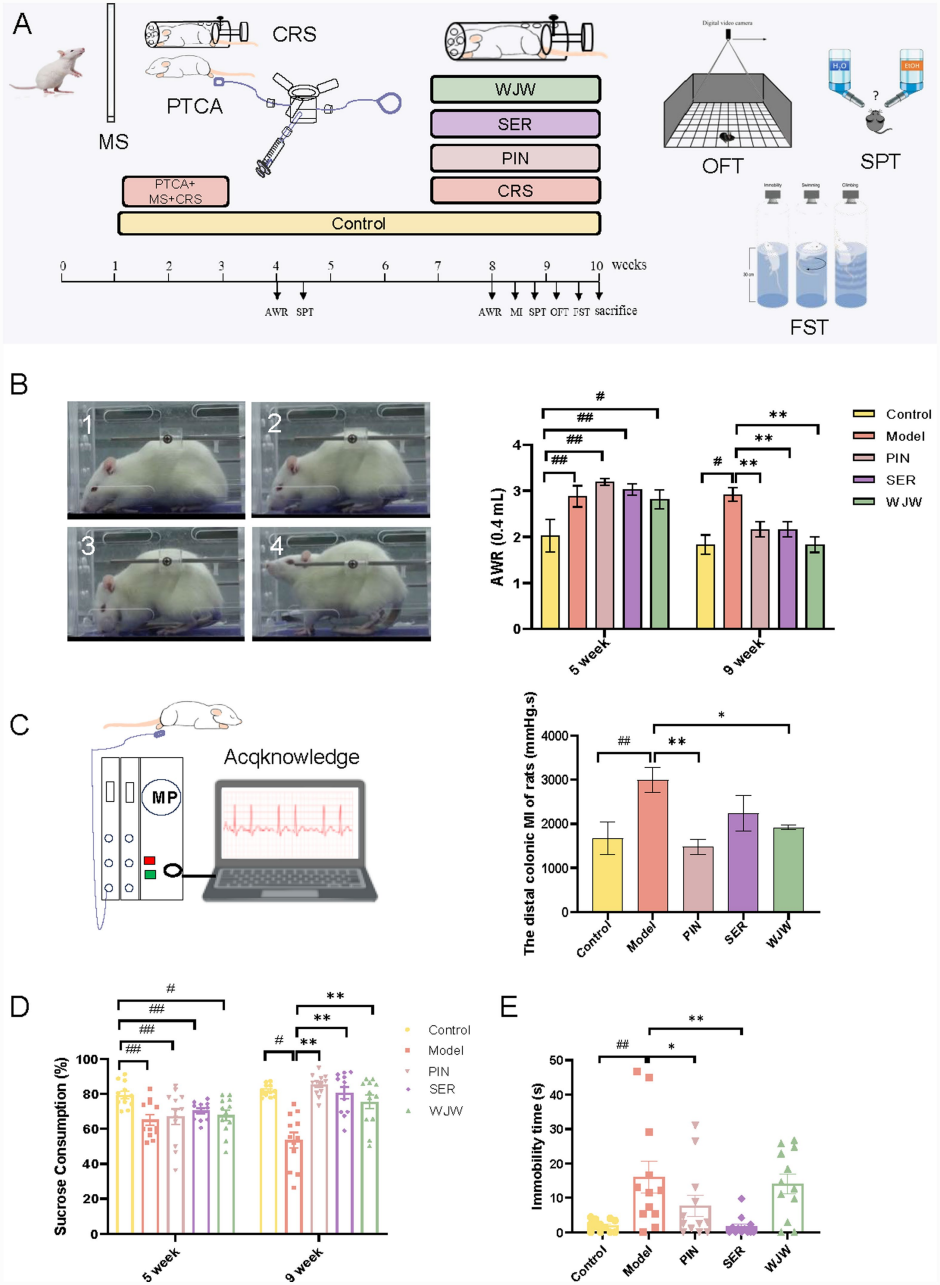
Wuji Pill ameliorates intestinal dysfunction and alleviates depression-like behaviors of IBS rats. **(A)** The preparation procedure and experimental design of the IBS rat model. **(B)** The schematic diagram and detection results of the AWR score (*n* = 12). 1 point, immobility and reduced head movement; 2 points, abdominal muscle contraction, but not lifted; 3 points, lifting the abdomen; 4 points, the body is arched and the perineum is lifted. **(C)** The detection results of the colon movement curve (*n* = 5). **(D)** Sucrose preference test (*n* = 12). **(E)** Forced swimming test (*n* = 12). Data are presented as the mean ± SEM, compared with the control group, # *p* < 0.05, *## p* < 0.01; compared with the model group, * p < 0.05, ** p < 0.01.

The experimental design 1 is shown in Figure 1A. The rats were randomly divided into five groups: control, model, pinaverium bromide (PIN), sertraline (SER) and Wuji Pill (WJW). The control and model groups were administered water. The treatments were as follows: (1) PIN group: the model was established and pinaverium bromide (13.5 mg/kg; 717,640; Abbott) was orally administered daily for 3 weeks; (2) SER group: the model was established and sertraline (20 mg/kg; EM4203; Pfizer) was orally administered daily for 3 weeks; and (3) WJW group: According to the previous administration plan (Yang et al., 2022), Wuji Pill (300.87 mg/kg) was orally administered daily for 3 weeks at a clinically equivalent dose.

#### 2.4.2 Antibiotic and Wuji Pill administration

For the antibiotic group (ABX), the model was established and Wuji Pill (300.87 mg/kg) was administered daily for 3 weeks, and drinking water containing four antibiotics was used to remove gut microbiota at the same time (Ren et al., 2018). The four antibiotics were streptomycin (1 g/L, MB1275, MeilunBio), ampicillin (1 g/L, MB1378, MeilunBio), gentamicin (1 g/L, MB1331, MeilunBio) and vancomycin (0.5 g/L, MB1260, MeilunBio).

#### 2.4.3 Fecal microbiota transplantation

For the FMT group, the model was established and the rats were given 2 mL fresh fecal bacteria solution orally administered daily for 3 weeks. The fresh fecal bacteria solution came from the fresh feces of rats three days after oral administration of Wuji Pill. Briefly, the collected feces were resuspended in normal saline 1:5 (w/v). Gauze was used to filter the suspension, and the supernatant was discarded after centrifugation (3,000 rpm/min, 10 min, 4 °C). Add normal saline again and repeat for 3 times to obtain fresh fecal bacteria solution.

#### 2.4.4 *Akkermansia muciniphila* administration

For the *A. muciniphila* group (AKK), the model was established and *A. muciniphila* (1 × 10^9^ CFU/d) were administered daily for 3 weeks. The culture of *A. muciniphila* was referred to the corresponding literature (Qin et al., 2018). Briefly, *A. muciniphila* freeze-dried powder (ATCC BAA-835, BNCC) was purchased and cultured in an anaerobic incubator (YQX-II, Shanghai Cimo Medical Instrument Co., Ltd., China) using BHI broth (CM917, Beijing Land Bridge Technology Co., Ltd., China). After plate counting, the bacteria were frozen with 30% glycerol. After thawing, the bacteria were used for intragastric administration.

### 2.5 Abdominal wall withdrawal reflex test

Abdominal wall withdrawal reflex test (AWR) was used to evaluate visceral sensitivity in rats. The catheter (Paraffin lubricated) was inserted into the rat anus for 4 cm and fixed at the root of the rat tail. After the rats were immobile, 0.4 or 0.6 mL of normal saline was injected into the catheter balloon. The visceral sensitivity of rats was scored according to the scoring principle of AWR experiment, as shown in Figure 1B (Al-Chaer et al., 2000).

### 2.6 Recording of distal colonic motility and calculation of motility index

The distal colonic motility was recorded to evaluate the intestinal motility of rats. After fasting overnight, the rats were fixed in the bonder. The catheter (Paraffin lubricated) was inserted into the rat anus for 4 cm and 0.4 mL of normal saline was injected into the catheter balloon. The area under the colonic motility curve (motility index, MI) was recorded after 30 min of adaptation (BIOPAC, MP150, physiograph; BIOPAC, Goleta, CA, United States), and the colonic motility was quantitatively described by MI, as shown in Figure 1C (Iwa et al., 2006).

### 2.7 Behavioral tests

#### 2.7.1 Sucrose preference test

The sucrose preference test (SPT) is used to evaluate the emotional state of animals with lack of motivation and pleasure (Verharen et al., 2023). First, the rats were trained for 48 h on the 30th day after birth, and fasted during the training. Rats were given two bottles of 1% sucrose solution in the first 24 h. After 24 h, a bottle of 1% sucrose solution and a bottle of purified water were given at the same time, and the positions of two bottles were exchanged three times during the period. Further, the SPT was performed on the 32nd and 61st day after birth. After fasting and water deprivation for 14 h in a single cage, rats were given a bottle of 1% sucrose solution and a bottle of purified water. Weigh and calculate the drinking volume of two bottles of solution within 2 h.

#### 2.7.2 Open field test

The open field test (OFT) is used to evaluate the autonomous behavior and exploratory behavior of animals in new and different environments (Kraeuter et al., 2019). Rats were placed in a quiet setting, gently positioned facing the inner wall of a 100 × 100 × 50 cm test box. The camera recorded the movement of rats within 5 min after they were put into the test box. After each trial, the box was cleaned with alcohol and water to avoid scent interference. The exercise time and the ratio of central area exercise distance were calculated by software (Smart 3.0, Panlab).

#### 2.7.3 Novelty-suppressed feeding test

The novelty-suppressed feeding test (NSFT) is a method to evaluate the intake interest and appetite of animals (Chevalier et al., 2020). The rats were placed in a quiet experimental environment and fasted for 12 h before the experiment. One grain of rat food is placed in the center of the test box (50 × 50 × 50 cm). The rats were gently placed towards the inner wall of the test box. Record the time starting from when the rats are placed into the test box until the moment they commence feeding.

#### 2.7.4 Forced swimming test

Forced swimming test (FST) is a method to evaluate the behavior and physiological changes of animals in stress environment (Yankelevitch-Yahav et al., 2015). On the day before the experiment, the animals were pre-swimming for 15 min (water temperature: 23 °C). On the day of experiment, the rats were placed in a transparent tube (diameter: 20 cm, water depth: 25 cm, water temperature: 23 °C) for 5 min. The immobility time of rats in the transparent bucket was recorded by software (Smart 3.0, Panlab).

### 2.8 Sample collection

To minimize animal suffering and distress, euthanasia was performed under anesthesia induced by isoflurane. Following the completion of the behavioral test, the rats were anesthetized using an experimental bench animal anesthesia ventilation system (Midmark) with a 3% concentration of inhaled isoflurane. Subsequently, euthanasia was performed under anesthesia by collecting blood via the abdominal aorta. The feces of rats were collected aseptically and stored at −80 °C for further analysis. Subsequently, the hippocampus and colon tissues were carefully cleaned in cold normal saline. Excess water was gently absorbed using filter paper, after which the tissues were promptly immersed in liquid nitrogen for cryopreservation. The samples were then transferred and stored at −80 °C for subsequent analysis. Cardiac perfusion was performed after anesthesia in rats, and the colon and brain tissues were fixed in 4% paraformaldehyde for histological analysis.

### 2.9 AB-PAS staining

The colon samples fixed in paraformaldehyde were embedded in paraffin and sliced. The colonic mucus secretion was detected by alcian blue periodic acid Schiff (AB-PAS) staining according to the protocol (Yamabayashi, 1987). The paraffin sections were dewaxed to distilled water and 3% acetic acid solution for 3 min. Then, the colon was stained according to the instructions of AB-PAS (CAS12040-44-7, Shanghai Tensus Biotech Co., Ltd.). Finally, the secretion of colonic mucus was observed under a microscope. ImageJ software was used to measure the percentage of acidic mucin area relative to the total tissue area.

### 2.10 Immunofluorescence staining

The morphology of microglia was detected by immunofluorescence staining according to the protocol (Im et al., 2019). Briefly, euthanasia was performed under anesthesia induced by isoflurane. The rats were anesthetized using an experimental bench animal anesthesia ventilation system (Midmark) and inhaled isoflurane at a concentration of 3% and subjected to cardiac perfusion. For the cardiac perfusion process, first perfuse with pre-cooled 0.9% normal saline to wash out the blood in the blood vessels. Subsequently, immediately perform perfusion fixation with pre-cooled 4% paraformaldehyde phosphate buffer solution (0.01 M PBS) (SparkJade). The brain sample was fixed in paraformaldehyde, then embedded in paraffin and sliced. Brain sections were dewaxed to water, antigen repair, hydrogen peroxide and serum blocking in turn. After washing off the sealing solution, the primary antibody (1:500, GB113502, Servicebio) was added and incubated at 4 °C overnight. After eluting, the secondary antibody (1:500, GB23301, Servicebio) was added and incubated for 50 min. After incubation with iF555-TSA (1:500, Servicebio, G1233), the sections were cleaned, microwave treated and sealed. DAPI (Servicebio, G1012) was used to stain the nuclei, and then the sections were rinsed and sealed.

### 2.11 RNA extraction and real-time fluorescent quantitative PCR

Total RNA from colon and hippocampus was extracted according to RNA Easy Fast Tissue/Cell Kit (DP451, Tiangen Biotech). The quantity and quality of RNA were performed by instrument (Biochrom SimpliNano, America). The total RNA was transcribed into cDNA according to EasyScript^®^ First-Strand cDNA Synthesis SuperMix (AE301, TransGen Biotech). Further, amplification was performed by PerfectStart^®^Green qPCR SuperMix procedure (AQ601, TransGen Biotech) on a real-time fluorescent quantitative PCR (LightCycler 480II, Roche). The primer sequence was shown in the Table 1. Calculate according to the average Ct of multiple holes. Using GAPDH as the internal reference gene.

**Table 1:** RT-PCR primer sequence.

Gene	Forward	Reverse	GenBank accession
TPH2	AGTCCTCATGTACGGCACCG	CTGGGAATGGGCTGGCCATA	NM_173839.3
TPH1	TACAATCCGTACACACAGAGCATTCAG	TAGCAAGGGCATCATTGACGACATC	NM_001100634.4
MAOA	GGAGGCGGCATCTCAGGATT	ATCCCGGGCTTCCAAAACCA	NM_033653.1
MAOB	TGCAGCCAGTCCATTATGAAGAGAAG	TCAAGATGCCAGGAGGGAAGTAGG	NM_013198.1
IDO1	AGCATCAAGACCCGAAAGCACTG	GATCCACGAAGTCACGCATCCTC	NM_023973.2
KMO	CGCATGTCAACTCTAGGTGGTTCC	GCCTCGTGGTATCTTATTCTGGTGAAG	NM_021593.2
QPRT	CATGTAGCAGGCACGAGGAAGAC	CAGGTCATAGCGGTGGCATTCAG	NM_001009646.1
GAPDH	AGTTCAACGGCACAGTCAAGGC	CGACATACTCAGCACCAGCATCAC	NM_017008.4

### 2.12 DNA extraction and real-time fluorescent quantitative PCR

The genomic DNA of *A. muciniphila* was extracted by TIANamp Bacteria DNA Kit (DP302-02, Tiangen Biotech). TIANamp Stool DNA Kit (DP328-02, Tiangen Biotech) was used to extract genomic DNA from stool samples of rats. The above operations are carried out in strict accordance with the instructions. Further, qPCR was performed on a real-time fluorescent quantitative PCR instrument (lightcycle 480 II, Roche) using SYBR green reagent (AQ601, TransGen Biotech). The gene specific primers of *A. muciniphila* were synthesized according to the literature (Qin et al., 2018). The average Ct of multiple holes is substituted into the standard curve for calculation.

### 2.13 16S rRNA sequencing

The 16S rRNA sequencing was used to detect the composition and relative abundance of gut microbiota. Briefly, DNA was extracted from fecal samples in strict accordance with the protocol in the instructions (Wang et al., 2025). The V3–V4 hypervariable region of the 16S rRNA was amplified using universal primers 341F (5’-CCTACGGGNGGCWGCAG-3′) and 805R (5’-GACTACHVGGGTATCTAATCC-3′). QIIME2 were used for sequences processing. Amplicon sequence variants (ASVs) were obtained after using DADA2 for dereplication and denoising. The raw data obtained after sequencing on the machine were subjected to subsequent bioinformatics analysis. This part of the sequencing experiment was assisted by Shanghai Zhongke New Life Biotechnology Co., Ltd. The Bray-Curtis distance was used for Principal coordinates analysis (PCoA). Alpha diversity analysis (including Chao1 index and Shannon index) was used to analyze the microbial differences. Linear discriminant analysis Effect Size (LEfSe) was used to identify differentially abundant taxa between groups. The raw sequence data were deposited in the Genome Sequence Archive in National Genomics Data Center (CNCB-NGDC Members and Partners, 2022), China National Center for Bioinformation/Beijing Institute of Genomics, Chinese Academy of Sciences (GSA: CRA023334).

### 2.14 Targeted metabonomics analysis

The metabolites in the hippocampus, colon and feces were analyzed using the AB SCIEX triple Quad ™ 6,500 LC–MS/MS system (AB Sciex, Canada). The hippocampus (about 30 mg) was added with 150 μL methanol: acetonitrile (v/v = 1:1) to prepare homogenate. Add 10 μL of the internal standard working solution to 100 μL of the homogenate, and then perform a centrifugation operation (13,000 rpm, 4 °C, 20 min). The supernatant was used for LC–MS/MS analysis. The colon (about 200 mg) was added with 1 mL methanol: acetonitrile (v/v = 1:1) to prepare homogenate. Add 20 μL internal standard working solution to 200 μL homogenate and centrifuge (13,000 rpm, 4 °C, 20 min). The supernatant (160 μL) was concentrated by vacuum centrifugation (37 °C, 45 min), then added 80 μL methanol: acetonitrile (v/v = 1:1) for centrifugation (13,000 rpm, 4 °C, 15 min). The supernatant was used for LC–MS/MS analysis. The fecal samples were prepared according to the method described previously (Wang et al., 2025).

For hippocampus and colon samples, Waters Acquity UPLC HSS PFP column (2.1 × 100 mm, 1.8 μm) was used for chromatographic separation. The mobile phase consisted of solvent A (0.1% formic acid water) and solvent B (0.1% formic acid acetonitrile) at 35 °C with a typical flow rate (0.3 mL·min-1). The elution gradient of mobile phase was as follows: 0–1.5 min, 2% B; 1.5–5 min, 2% B -98% B; 5–7 min, 98% B; 7.1–10 min,2% B. Multiple reaction monitoring mode (MRM) and cationic electrospray ion source (ESI+) were used. Other parameters were as follows: ion source spray voltage, 4,500 V; inlet voltage, 10 V; impact chamber outlet voltage, 11 V; air curtain pressure, 35 psi; collision pressure, 9 psi; ion source temperature: 450 °C. For fecal samples, Waters ACQUITY UPLC HSS T3 column (2.1 × 100 mm, 1.8 μm) was used for chromatographic separation. The mobile phase consisted of solvent A (0.1% formic acid water) and solvent B (isopropanol) at 50 °C with a typical flow rate (0.2 mL·min^−1^). The elution gradient of mobile phase was as follows: 0–1 min 15% B; 1–8 min 15% B-36% B; 8–8.5 min 36% B-50% B; 8.5–10.5 min 50% B; 10.5–10.51 min 50% B-15% B; 10.51–15 min 15% B. Other parameters were set as mentioned above. Analytical parameters for target ion pairs were summarized in Tables 2, 3.

**Table 2:** The quantitative analysis parameters of tryptophan metabolites.

Name	Q1 (m·z^−1^)	Q3 (m·z^−1^)	DP (V)	CE (V)
Tryptophan	205.1	188.0	200	9
5-hydroxytryptamine	177.0	160.0	80	10
5-hydroxyindoleacetic acid	192.0	146.0	120	10
N′-Formylkynurenine	237.0	192.1	34	10
Kynurenine	209.0	192.0	90	8
3-hydroxykynurenine	225.0	109.8	80	10
Quinolinic acid	168.2	95.1	45	10
Isoprenaline (IS)	212.2	194.2	20	10

**Table 3:** The quantitative analysis parameters of SCFAs.

Name	Q1 (m·z^−1^)	Q3 (m·z^−1^)	DP (V)	CE (V)
Acetic acid	166.1	91.2	50	18
Propionic acid	180.1	91.2	68	20
Butyric acid	194.2	91.2	72	21
Isobutyric acid	194.2	91.1	89	20
Acetic acid C-13 (IS)	167.1	91.2	91	59

### 2.15 Statistical analysis

Statistical analyses were performed with GraphPad Prism 9 (La Jolla, CA, United States). All data of the experimental results were expressed by mean ± SEM. The data of each group were processed by one-way ANOVA or Kruskal-Wallis test. Fisher’s LSD test or Dunn’s post-hoc test was used for multiple comparison analysis. *P*-value < 0.05 was considered statistically significant.

## 3 Results

### 3.1 Wuji Pill improves intestinal symptoms and depression-like behavior in IBS rats

In order to simulate the symptoms of gastrointestinal motility disorders accompanied by depression in IBS patients, we successfully established an animal model of IBS by PTCA balloon stimulation, MS and CRS. The procedure was shown in Figure 1A. The experimental results demonstrated that IBS model rats exhibited significantly higher AWR scores compared to the control group, indicating that two stages stimulation protocols effectively induced visceral hypersensitivity in the intestinal tract (Figure 1B; Supplementary Figure S3A), and the colonic motility index was significantly increased (Figure 1C). Furthermore, the exercise time of IBS rats did not change (Supplementary Figure S3B), and the sucrose consumption significantly decreased in both the juvenile and adult stages (Figure 1D). Meanwhile, the immobility time in the FST of IBS rats significantly increased (Figure 1E).

At the same time, pinaverium bromide (antispasmodic) and sertraline (antidepressant) were employed as positive drugs, which are commonly used to improve IBS in clinic. Pinaverium bromide can significantly reduce the AWR score and colonic motility index in IBS rats, the sucrose consumption was significantly increased and the immobility time in the FST was decreased, which plays a certain role in improving depressive-like behaviors. Sertraline can significantly increase the sucrose consumption and reduce the immobility time in the FST. Wuji Pill can significantly improve the symptoms of abdominal pain and colonic motility in rats (Figures 1B,C). Furthermore, the treatment with Wuji Pill remarkably ameliorated the depression-like behaviors in IBS rats. The IBS rats administered with Wuji Pill exhibited an augmented sucrose consumption (Figure 1D), and alleviated the desperate state of rats in a stressful environment (Figure 1E). These results indicate that Wuji Pill has the potential to improve the intestinal symptoms and depression-like behaviors of IBS rats.

### 3.2 Wuji Pill modulates the gut microbiota composition in IBS rats

**Figure 2:**
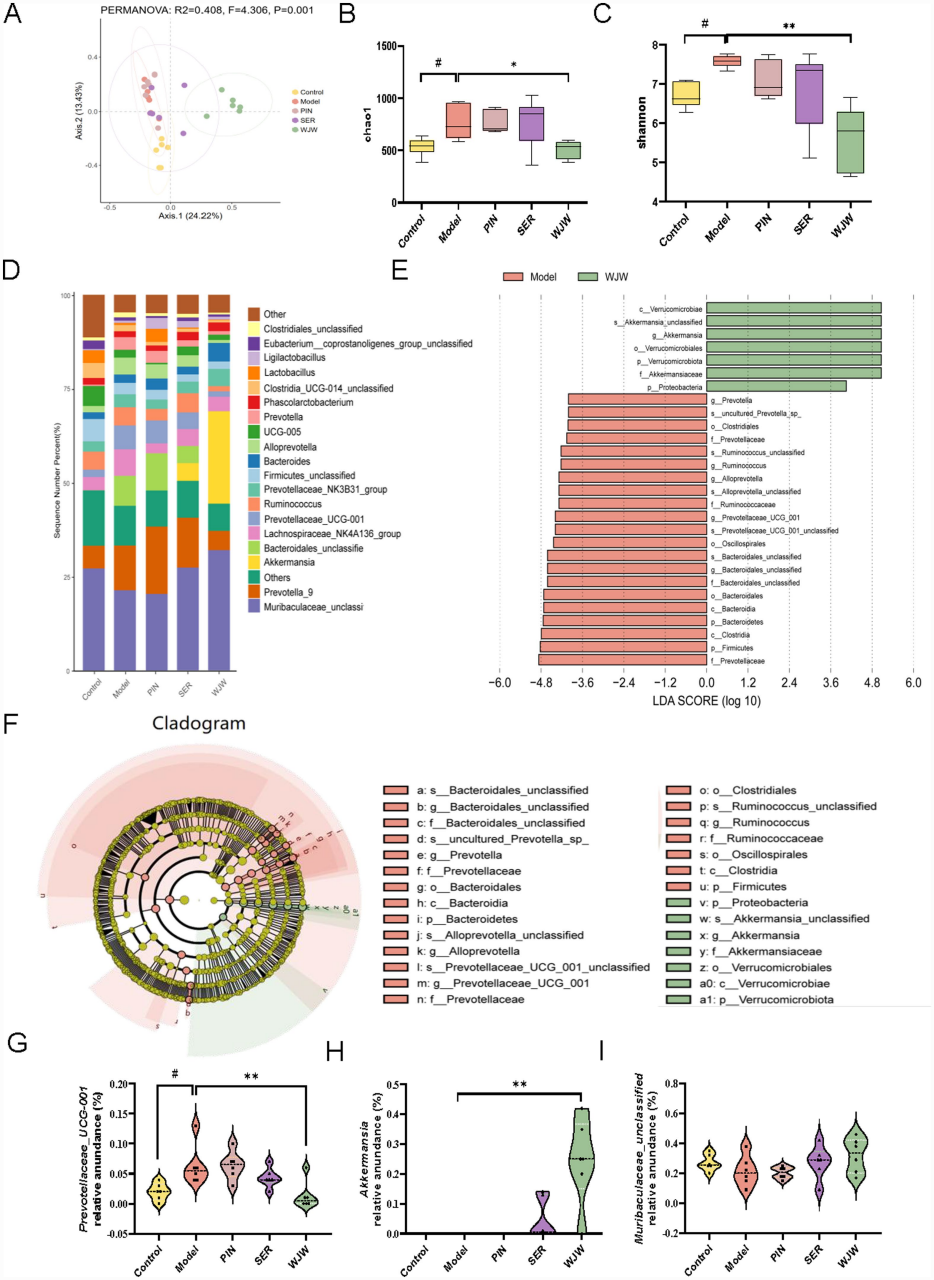
Wuji Pill modulates the gut microbiota of IBS rats. **(A)** Principal coordinate analysis (PCoA) of the Bray-Curtis distance. **(B,C)** Alpha diversity analysis. **(D)** Analysis of the gut microbiota taxon composition. **(E,F)** Gut microbiota LEfSe analysis. **(G–I)** The relative abundance of *Prevotellaceae_UCG-001* **(G)**, *A. muciniphila* **(H)**, and *Muribaculaceae_unclassified* **(I)**. Data in **(B,C)** are presented as box-and whisker plots (Mix-Max), in which the horizontal line represents the median, the box indicates the interquartile range, and the whiskers extend to the minimum and maximum values. Compared with the control group, # *p* < 0.05; compared with the model group, ** *p* < 0.01.

In this study, 16S rRNA gene sequencing technology was used to evaluate changes in the gut microbiota of rats. Based on the principal coordinate analysis (PCoA) of Bray-Curtis distance, we revealed that the gut microbiota composition of the model group was significantly different from that of the control group and Wuji Pill group (Figure 2A). Chao1 index and Shannon index belong to alpha diversity analysis, representing the abundance and diversity of taxon composition, respectively (Shi et al., 2023). The Chao1 index of IBS rats increased significantly (Figure 2B), and the Chao1 index and Shannon index decreased significantly after oral administration of Wuji Pill (Figures 2B,C). In addition, the taxon composition results showed that *Bacteroidota* and *Firmicute* were the dominant bacteria at the phylum level (Supplementary Figure S3C). Further, *Prevotella* was the main difference between the control group and the model group (Supplementary Figure S3D). *Prevotellaceae*, *Muribaculaceae*, *Firmicutes* and *A. muciniphila* were the main difference bacteria between the model group and the Wuji Pill group (Figure 2D). The relative abundance of *Prevotellaceae_UCG-001* in the model group increased significantly (Figures 2E,G). Interestingly, the currently highly regarded next-generation probiotic *A. muciniphila* significantly increased in the Wuji Pill group (Figures 2F,H). *Muribaculaceae* showed an increasing trend in the Wuji Pill group (Figure 2I). In short, the composition of the gut microbiota was significantly changed after treatment with Wuji Pill.

### 3.3 Wuji Pill improves intestinal mucus secretion in IBS rats through gut microbiota

**Figure 3:**
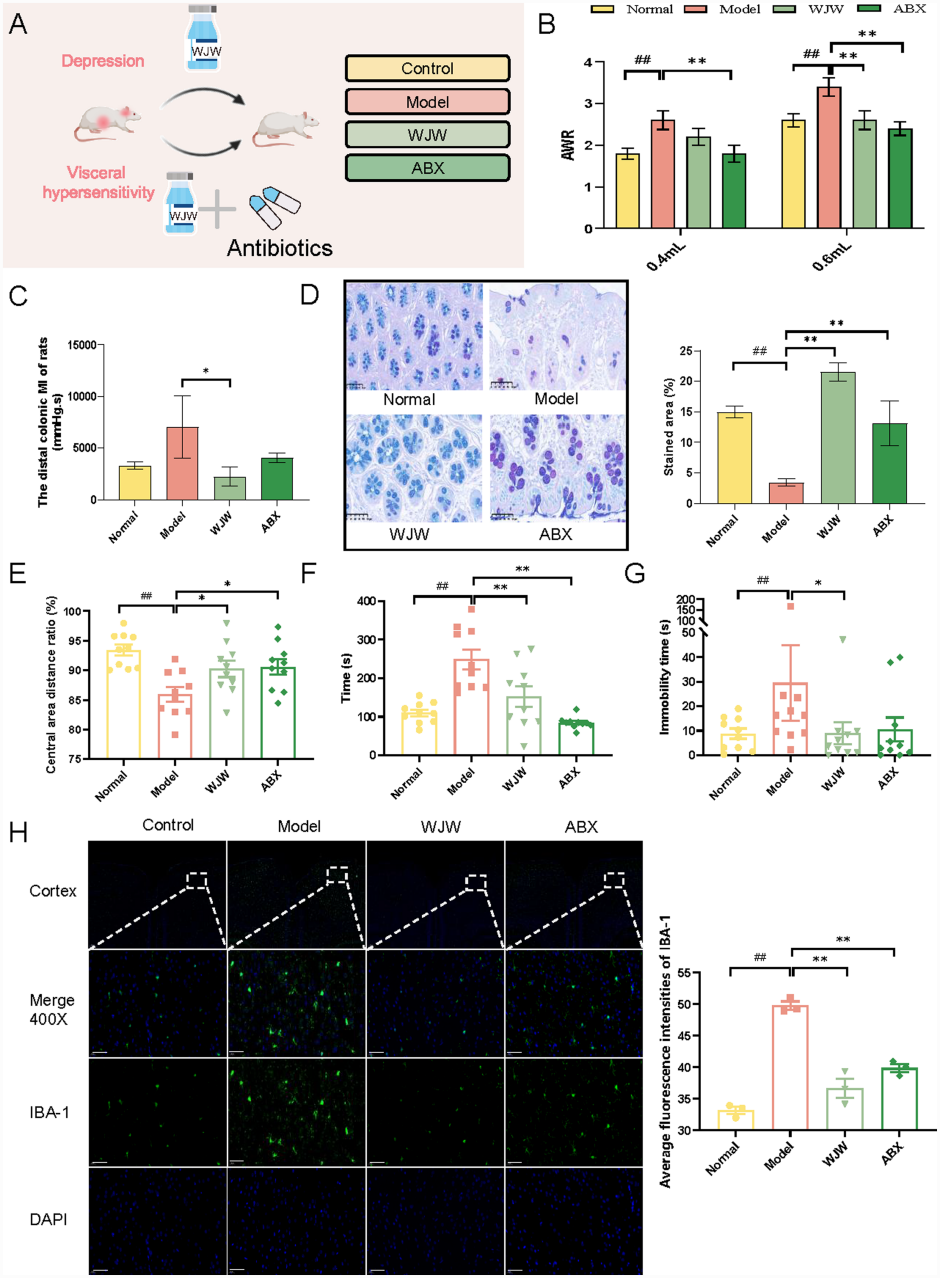
Wuji Pill ameliorates intestinal mucus secretion dysfunction and depression-like behaviors in IBS rats via gut microbiota. **(A)** Establishment of the pseudo germ-free model and experimental design. **(B)** The results of the AWR score (*n* = 10). **(C)** The detection results of the colon movement index (*n* = 3). **(D)** AB-PAS staining area of acidic mucin (*n* = 3). **(E)** Open field test (*n* = 10). **(F)** Novelty-suppressed feeding test (*n* = 10). **(G)** Forced swimming test (*n* = 10). **(H)** Cortical IBA-1 immunofluorescence staining (*n* = 3, scale bars = 50 μμm). Data are presented as the mean ± SEM, compared with the control group, *## p* < 0.01; compared with the model group, * p < 0.05, ** p < 0.01.

A germ-free animal model is often used to explore the relationship between gut microbiota and the host. We established a pseudo germ-free model by using mixed antibiotics to explore the improvement effect of Wuji Pill on IBS (Figure 3A). Wuji Pill also improved intestinal visceral sensitivity (Figure 3B) and reduced intestinal motility index (Figure 3C) in ABX group. Intestinal mucus constitutes a crucial component of the intestinal barrier (Aburto and Cryan, 2024). Goblet cells and other secretory cells play an important role in the secretion of intestinal mucus (Birchenough et al., 2015). The results showed that the secretion of mucus in the colon of IBS rats was significantly reduced (Figure 3D). Notably, oral administration of Wuji Pill significantly ameliorated this colonic mucus depletion. Furthermore, antibiotic intervention induced a marked reduction in intestinal mucus secretion (Figure 3D). These results indicate that gut microbiota plays an essential role in maintaining mucosal secretory homeostasis.

We further investigated the improvement effect of Wuji Pill on depressive-like behaviors in pseudo germ-free IBS rats. Similarly, the ABX group also has the effect of improving the depressive-like behavior of IBS rats. In the ABX group, the distance of movement in the central area of the OFT increased (Figure 3E). Moreover, both the time of NSFT and the immobility time of FST were shortened (Figures 3F,G). In addition, IBA-1, as a marker of microglia, reflects health and activation status of microglia (Lituma et al., 2021). Compared with the control group, the expression of IBA-1 in the cortex of the model group was significantly up-regulated. Notably, Wuji Pill was able to downregulate the expression of IBA-1 (Figure 3H).

### 3.4 Effects of Wuji Pill on gut microbiota and fecal metabolites in IBS rats

**Figure 4:**
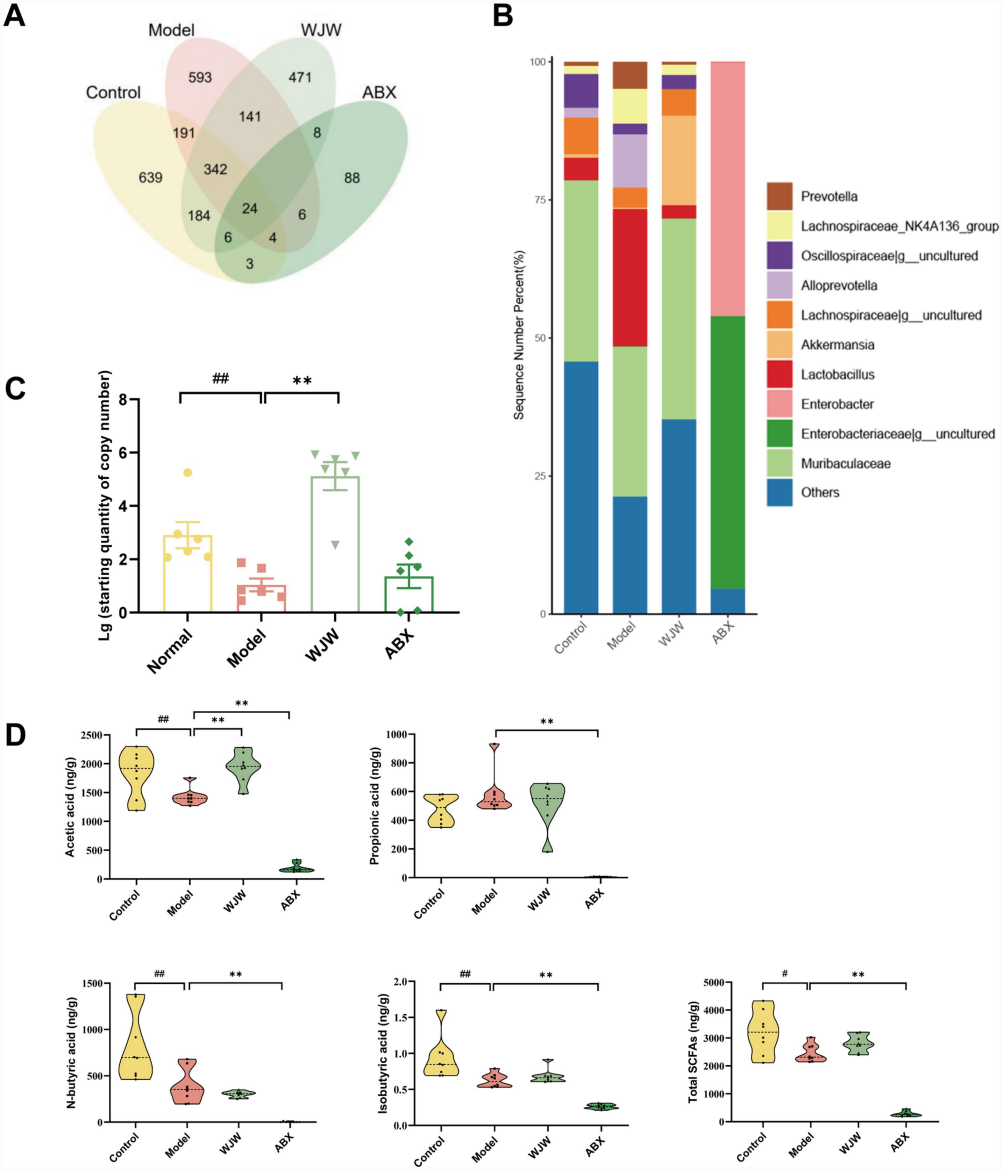
Wuji Pill affects the levels of SCFAs of IBS rats. **(A)** Venn diagram. **(B)** Analysis of the gut microbiota taxon composition (*n* = 6). **(C)** Absolute quantitative detection of *A. muciniphila* (*n* = 6); **(D)** The level of short-chain fatty acids in feces (*n* = 8). Data are presented as the mean ± SEM, compared with the control group, *^##^ p* < 0.01; compared with the model group, ** p < 0.01.

16S rRNA gene sequencing technology and real-time fluorescent quantitative PCR technology were used to compare the changes of gut microbiota. The results demonstrated that antibiotic intervention significantly reduced the diversity of gut microbiota (Figures 4A,B). The absolute abundance of *A. muciniphila* in Wuji Pill group was significantly greater than that in model group, and *A. muciniphila* in feces was significantly removed by antibiotics (Figure 4C). The results of LC–MS/MS indicated that the levels of acetic acid, propionic acid and total short-chain fatty acids (SCFAs) in the feces of IBS rats were decreased (Figure 4D). Wuji Pill could significantly reverse the level of acetic acid (Figure 4D). After the administration of antibiotics, the levels of SCFAs in feces decreased significantly (Figure 4D).

### 3.5 FMT and *Akkermansia muciniphila* alleviate intestinal dysfunction and depression-like behavior in IBS rats

**Figure 5:**
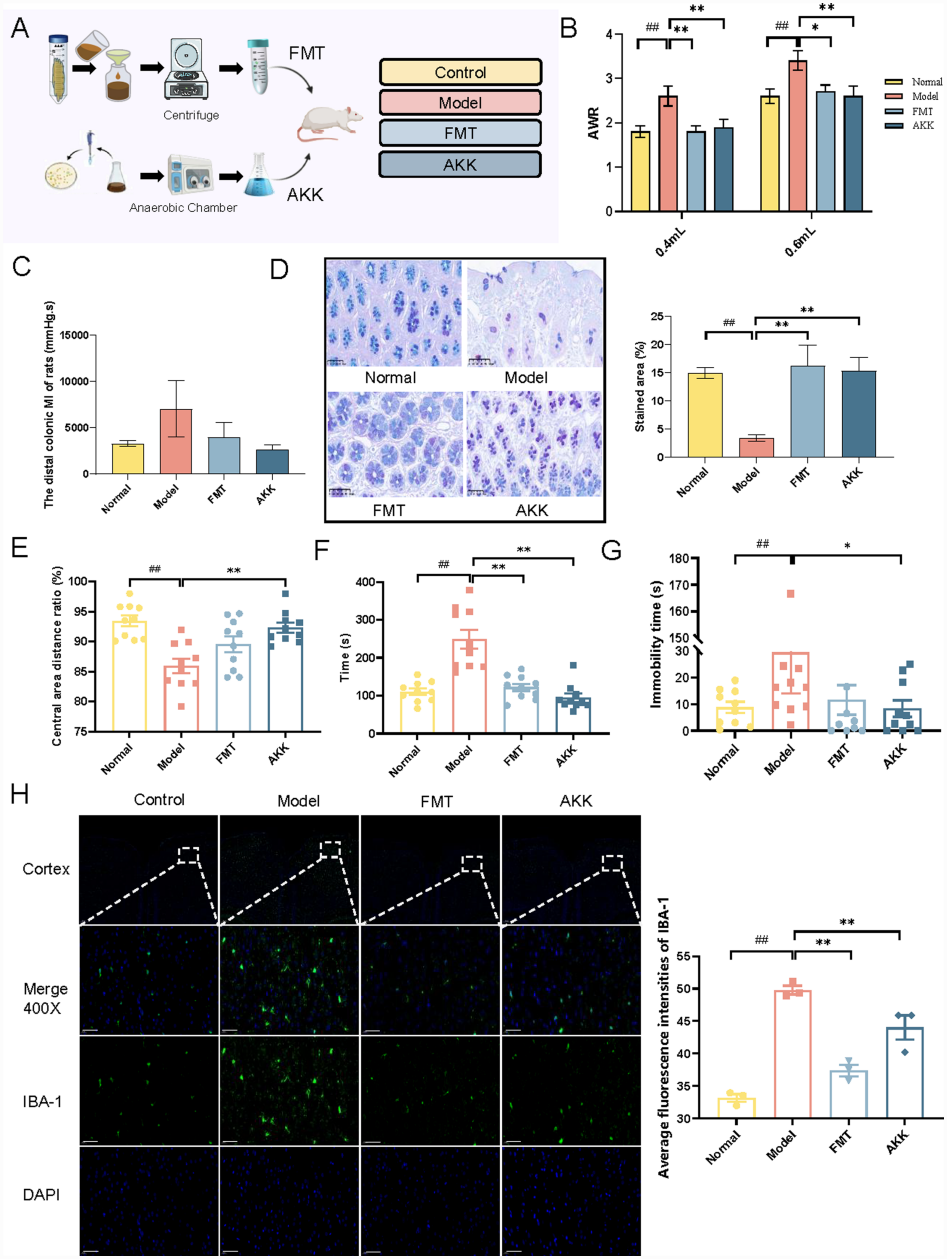
Microbiota transplantation ameliorates intestinal dysfunction and alleviates depression-like behaviors of IBS rats. **(A)** Microbiota transplantation operation schematic diagram and experimental design. **(B)** The results of the AWR score (*n* = 10). **(C)** The detection results of the colon movement index (*n* = 3). **(D)** Colon mucus staining (*n* = 3). **(E)** Open field test (*n* = 10). **(F)** Novelty-suppressed feeding test (*n* = 10). **(G)** Forced swimming test (*n* = 10). **(H)** Cortical IBA-1 immunofluorescence staining (*n* = 3, scale bars = 50 μm). Data are presented as the mean ± SEM, compared with the control group, *^##^ p* < 0.01; compared with the model group, * p < 0.05, ** p < 0.01.

In the subsequent experiment, intragastric administration was used for microbiota transplantation and the improvement of gut microbiota on IBS was evaluated. Microbiota transplantation includes fecal microbiota transplantation (the gut microbiota following the intervention of Wuji Pill) and *A. muciniphila* (the probiotics significantly increased after Wuji Pill intervention) (Figure 5A). Compared with the model group, the visceral sensitivity of IBS rats was significantly reduced after the intervention of FMT and *A. muciniphila* (Figure 5B). In addition, abnormal colonic motility was relieved in IBS rats (Figure 5C). Meanwhile, the secretion of colonic mucus was significantly restored by the fecal microbiota and *A. muciniphila* (Figure 5D).

After the intervention of FMT and *A. muciniphila*, the ratio of distance in the central area (Figure 5E) and the exploration interest (Figure 5F) were significantly increased of IBS rats. The immobility time of FST was reduced (Figure 5G). It is further verified that FMT and *A. muciniphila* intervention can improve the depression-like behavior of IBS rats. In addition, the microglia in cortex were significantly inhibited after the intervention of FMT and *A. muciniphila* (Figure 5H).

### 3.6 FMT and *Akkermansia muciniphila* transplantation elevate the levels of SCFAs in IBS rats

**Figure 6:**
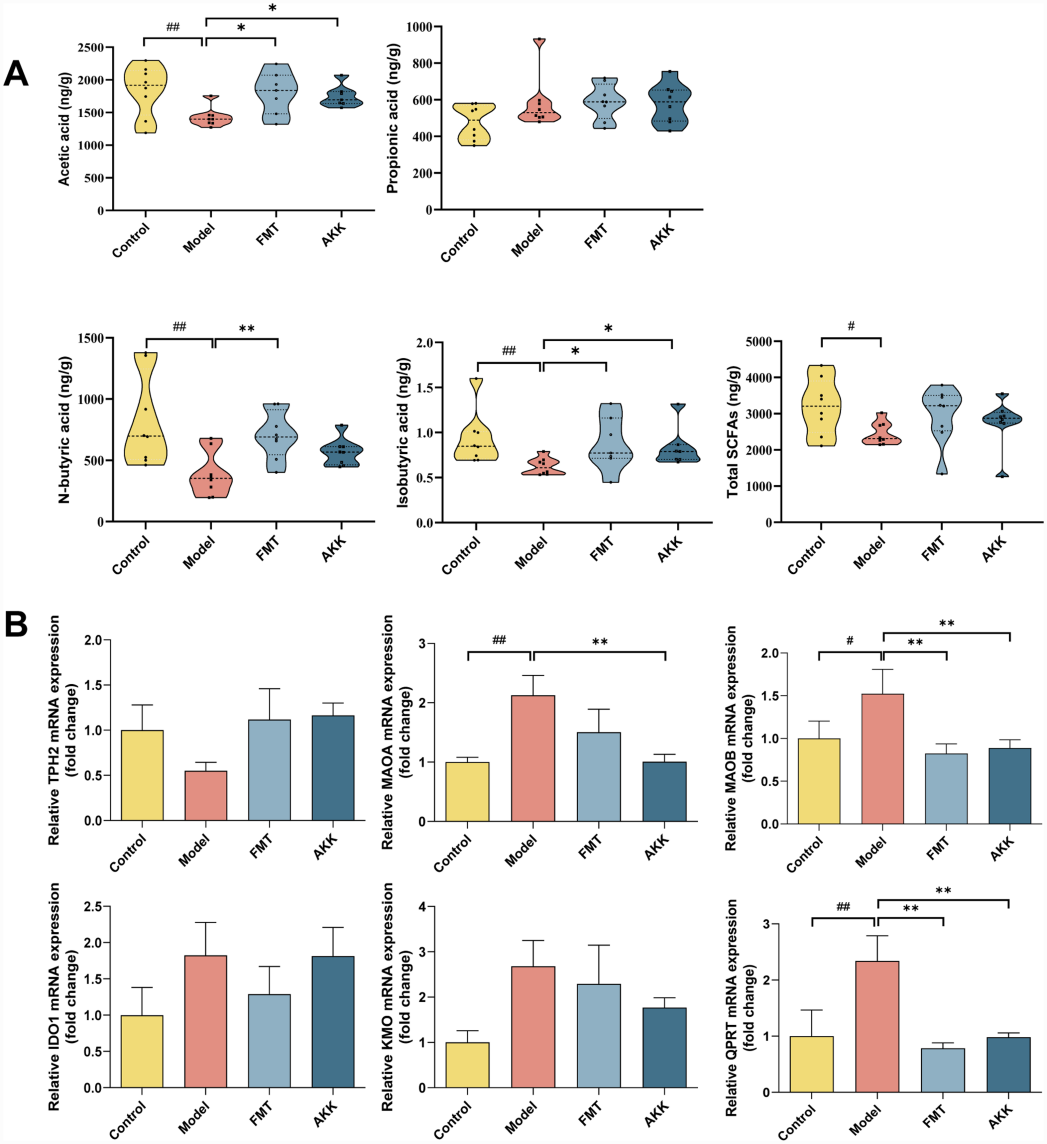
Microbiota transplantation affects the levels of SCFAs and the enzymes in the tryptophan metabolism pathways. **(A)** The level of short-chain fatty acids in feces (*n* = 8). **(B)** The expression of tryptophan metabolic enzymes in hippocampal (*n* = 6). Data are presented as the mean ± SEM, compared with the control group, *# p* < 0.05, *## p* < 0.01; compared with the model group, * p < 0.05, ** p < 0.01.

The changes of SCFAs in feces after intervention of microbiota were verified. The levels of acetic acid, n-butyric acid and isobutyric acid were significantly increased in FMT group (Figure 6A). *A. muciniphila* intervention significantly increased the levels of acetic acid and isobutyric acid (Figure 6A). These findings demonstrate that microbiota intervention directly influences the levels of SCFAs.

### 3.7 FMT and *Akkermansia muciniphila* transplantation influenced the tryptophan metabolic pathway within the gut-brain axis in IBS rats

The levels of tryptophan pathway metabolites in the hippocampus and colon of IBS rats after the intervention of gut microbiota were summarized in Table 4. In the hippocampus of IBS rats, the level of 5-hydroxytryptamine (5-HT) was significantly decreased and the levels of 3-hydroxykynurenine (3-HK) and quinolinic acid (QA) were significantly increased. After the intervention of FMT, 5-HT was significantly increased, and the levels of 3-HK were significantly reversed. 5-hydroxyindoleacetic acid (5-HIAA) was significantly decreased after *A. muciniphila* intervention. In the colon, 5-HT levels were increased in the model group. After FMT, the levels of tryptophan and 5-HIAA were significantly increased. 5-HT was significantly decreased after *A. muciniphila* intervention.

**Table 4:** Concentrations of tryptophan and the related metabolites in the hippocampus and colon (ng/mg).

Tissue	Compounds	Control	Model	FMT	AKK
Hippocampus	TRP	8.4024 ± 0.6773	7.8732 ± 0.4043	7.7565 ± 0.3556	7.6531 ± 0.5826
	5-HT	0.6434 ± 0.045	0.4631 ± 0.0306##	0.6268 ± 0.0306**	0.5001 ± 0.0276
	5-HIAA	0.3381 ± 0.0336	0.3717 ± 0.0322	0.366 ± 0.0136	0.2646 ± 0.0085**
	NFK	0.003 ± 0.0004	0.0038 ± 0.0003	0.0022 ± 0.0004**	0.0034 ± 0.0004
	KYN	0.072 ± 0.0054	0.0919 ± 0.0069	0.0616 ± 0.0077	0.0865 ± 0.0219
	3-HK	0.0898 ± 0.0079	0.1235 ± 0.0076##	0.0772 ± 0.0065**	0.1195 ± 0.0081
	QA	0.6747 ± 0.0324	0.9985 ± 0.0986#	1.219 ± 0.1302	0.8336 ± 0.0758
Colon	TRP	5.3346 ± 0.1519	4.4973 ± 0.1798#	6.0557 ± 0.3914**	5.1569 ± 0.1908
	5-HT	1.0425 ± 0.1736	1.4553 ± 0.1916	1.1104 ± 0.1913	0.7465 ± 0.0837*
	5-HIAA	0.0956 ± 0.0135	0.0906 ± 0.0094	0.1498 ± 0.0203**	0.1098 ± 0.0076
	NFK	0.014 ± 0.0028	0.0082 ± 0.0009#	0.0124 ± 0.0014	0.0087 ± 0.0008
	KYN	0.1061 ± 0.0081	0.0484 ± 0.0063##	0.0505 ± 0.0049	0.045 ± 0.004
	3-HK	0.0038 ± 0.0007	0.0054 ± 0.0008	0.0056 ± 0.0016	0.0044 ± 0.0009
	QA	0.3355 ± 0.021	0.3759 ± 0.0184	0.5125 ± 0.0198**	0.434 ± 0.0122

Compared with the control group, # *P* < 0.05, ## *P* < 0.01; compared with the model group, * *P* < 0.05, ** *P* < 0.01 (*n* = 8, mean ± SEM). TRP, Tryptophan; 5-HT, 5-hydroxytryptamine; 5-HIAA, 5-hydroxyindoleacetic acid; NFK, N′-Formylkynurenine; KYN, Kynurenine; 3-HK, 3-hydroxykynurenine; QA, Quinolinic acid.

Tryptophan metabolizing enzymes are the key points for regulating the tryptophan metabolic pathway. Further, we detected the levels of tryptophan metabolizing enzymes in the hippocampus and colon of IBS rats after the intervention of FMT and *A. muciniphila* (Figure 6B; Supplementary Figure S4). In the hippocampus of IBS rats (Figure 6B), the mRNA expression levels of MAOA, MAOB, and QPRT were significantly elevated. Conversely, in the FMT group, the mRNA expression levels of MAOB and QPRT were notably reduced. In the AKK group, the mRNA expression levels of MAOA, MAOB, and QPRT exhibited a reversal trend. In the colon (Supplementary Figure S4), the mRNA expression levels of THP1, MAOB and IDO1 increased in the model group. In the FMT group, the mRNA expression levels of THP1 and IDO1 decreased. Moreover, the level of IDO1 mRNA was decreased after *A. muciniphila* intervention.

## 4 Discussion

In this study, we reported the improvement effect of TCM prescription Wuji Pill on IBS combined with depression rat model. This study found that Wuji Pill can improve intestinal function and depression-like behavior in IBS rats. Furthermore, it was worth noting that *A. muciniphila* increased significantly after Wuji Pill intervention. Next, we found that FMT and *A. muciniphila* can improve intestinal function and depression-like behavior in IBS rats and increase the secretion of colonic mucus. The mechanism of Wuji Pill improving IBS may depend on regulating gut microbiota, increasing the level of acetic acid and butyric acid in feces, and regulating the tryptophan metabolic pathway of brain-gut axis. In general, these findings strongly support that Wuji Pill can improve IBS through MGB axis.

In this study, we established an IBS rat model with gut-brain function dysfunction. Specifically, we used PCTA balloon stimulation to induce visceral sensitivity in rats (Gong et al., 2022). Meanwhile, MS and CRS were used to simulate mental stress (Reagan et al., 2004; Yi et al., 2017). As in previous studies (Ford et al., 2020), IBS rats showed high visceral sensitivity and colonic motor dysfunction, and the intestinal mucus secretion was severely damaged. Behavioral assessments including the SPT and FST demonstrated that IBS model rats exhibited significant depression-like behaviors. Studies have shown that stress can cause activation of microglia (Tillmon et al., 2024). Abnormal activation of microglia may release excessive inflammatory mediators and neurotransmitters, thereby aggravating the symptoms of depression (Wu and Zhang, 2023). In the results of immunofluorescence staining, cortical microglia in IBS rats were significantly activated. In addition, the gut microbiota and its metabolites also exhibited evident disorder. SCFAs, as important metabolites of gut microbiota, can maintain the intestinal barrier function and regulate the immune and metabolic functions of the host. The level of total SCFAs in the feces of IBS patients was lower than that of healthy people (Ju et al., 2024). In our study, SCFAs in the feces decreased significantly. Additionally, the relative abundance of *Prevotellaceae* in fecal samples exhibited a significant increase. *Gammaproteobacteria*, *Prevotellaceae*, *Lachnospiraceae UCG004* may increase the risk of IBS (Zhang et al., 2024). *Prevotellaceae* helps break down carbohydrates, but it can act as an opportunistic pathogen and cause problems such as intestinal inflammation (Chen et al., 2021). Furthermore, the relative abundance of the beneficial bacterium *A. muciniphila* is significantly increased in IBS rats. The abundance of *A. muciniphila* in IBS rat model and IBS patients is significantly reduced (Ottman et al., 2017). In conclusion, the establishment of animal models simulating clinical patients provides an important guarantee for the exploration of disease diagnosis and treatment strategies.

It has been reported that the regulation of MGB axis has potential therapeutic effect on gastrointestinal and nervous system diseases (Zhao et al., 2024). As a TCM commonly used in gastrointestinal diseases, studies have proved that Wuji Pill can improve IBD by targeting the gut microbiota tryptophan metabolite indole-3-acetic acid to activate Aryl Hydrocarbon Receptor pathway (Jing et al., 2025). In terms of improving the nervous system, paeoniflorin in *Paeonia lactiflora* Pall is metabolized into benzoic acid by carboxylic acid esters, which enters the brain and is convert into D-serine to play an antidepressant effect (Zhao et al., 2018). Our previous research demonstrated that Wuji Pill can ameliorate symptoms in rat models of inflammatory irritable bowel syndrome (Chen et al., 2017). Research in inflammatory bowel disease models also indicates the anti-inflammatory effect of Wuji Pill. Wuji Pill was able to reduce the level of the TNF-*α* mRNA in the colons of mice (Jing et al., 2024). In the current study, behavioral tests confirmed that Wuji Pill can improve the depressive symptoms of IBS rats. Simultaneously, Wuji Pill also significantly regulate the gut microbiota and its metabolites. After administration of Wuji Pill, the content of acetic acid in feces was significantly increased after administration of Wuji Pill. Moreover, after administration of Wuji Pill, the relative abundances of *Prevotellaceae*, *Muribaculaceae, Firmicutes* and *A. muciniphila* exhibited notable changes. *A. muciniphila* is a bacterium that lives on mucus secreted by intestinal epithelium. *A. muciniphila* was significantly increased after the intervention of Wuji Pill. As in previous studies (Ioannou et al., 2025), *A. muciniphila* was negatively correlated with *Prevotella*. After administration of Wuji Pill, the relative abundance of *Prevotellaceae* was significantly reduced. In addition, studies have shown that the relative abundance of *A. muciniphila* is positively correlated with *Muribaculaceae*. In conclusion, the interactions among gut microbiota warrant further exploration. Therefore, it is necessary to further study the microbial ecological environment and microbial interactions after oral administration of Wuji Pill.

Furthermore, we investigated the effects of Wuji Pill on pseudo germ-free IBS rats. Our results showed that Wuji Pill improved the intestinal symptoms and depression-like behaviors of pseudo germ-free IBS rats. The use of a broad-spectrum antibiotic cocktail to establish a pseudo germ-free model served to eliminate the background influence of the gut microbiota. This is a well-established method for depleting gut microbiota in animal models, allowing us to specifically test whether the efficacy of Wuji Pill depends on the presence of gut microbiota. Importantly, there is a complex relationship between antibiotics and diseases. In the short term, the use of antibiotics has a certain anti-inflammatory effect and can regulate intestinal dysfunction. In clinical practice, the antibiotic rifaximin is used to improve the symptoms of diarrhea and abdominal pain in IBS (Deljavan Ghodrati and Comoglu, 2024). Short-term antibiotic exposure can provide temporary relief from diarrhea and abdominal pain in IBS by reducing microbial load and altering community structure. However, antibiotics can also cause negative effects such as the weakening of probiotic colonization and the generation of drug-resistant bacteria (Karakan et al., 2021). Meanwhile, the deficiency of metabolites such as short-chain fatty acids (SCFAs) produced by gut microbiota can disrupt the intestinal barrier, increase the risk of infection, and lead to diseases (Ikeda et al., 2022). Moreover, the use of antibiotics can also disturb the nervous system. The mucus secretion serves as a physical barrier to protect against foreign pathogenic microorganisms, playing a crucial role in maintaining the health and function of mucosal tissues (Aburto and Cryan, 2024). Based on our experimental results, where the increase in intestinal mucus secretion following Wuji Pill administration was lower in the pseudo-germ-free model compared to the group receiving Wuji Pill alone, we posit that the action of Wuji Pill is at least partially dependent on an intact gut microbiota. Although our results showed that short-term antibiotic use had no significant impact on the nervous system of IBS rats, whether there are other negative effects after long-term intervention requires more experiments to explore. Future studies will incorporate an “antibiotics-only in the IBS model” control group and employ aseptic animal models to further validate and elucidate the specific role of the gut microbiota in the therapeutic mechanism of Wuji Pill.

Wuji Pill can significantly regulate the structure and abundance of the gut microbiota. To explore the specific role of the gut microbiota regulated by Wuji Pill in improving IBS, FMT and *A. muciniphila* transplantation were used to verify the hypothesis. First of all, the selection of donors is crucial for the treatment of microbiota transplantation, and the therapeutic effect may be related to the donor microbiome (Hamazaki et al., 2022). The microbiota transplantation experiment included FMT and *A. muciniphila*. Secondly, increasing evidence indicates that FMT can implant beneficial bacteria to restore the intestinal mucosal barrier in IBS patients (Halkjær et al., 2018; He et al., 2022). FMT can restore gut microbiota diversity and function, and regulate the brain-gut axis to improve IBS (Lo et al., 2024). The results show that FMT and *A. muciniphila* can improve the intestinal function and depressive-like behaviors in IBS rats. The microbiota intervention can promote the secretion of intestinal mucus in IBS rats. In addition, the increase of SCFAs can provide energy for intestinal epithelial cells and improve nervous system function through brain-gut axis (Mathias et al., 2024). FMT significantly regulated the levels of acetic acid and butyric acid in the feces of IBS rats. Acetic acid can regulate intestinal pH and contribute to the growth of beneficial bacteria. Butyric acid has been widely studied in the protection of intestinal mucosal barrier and anti-inflammatory (Najafi et al., 2017). *A. muciniphila* supplementation can increase the levels of acetic acid and isobutyric acid. In summary, finding that the levels of acetic acid, n-butyric acid, and isobutyric acid were significantly decreased in the IBS rat model, we found that FMT and *A. muciniphila* transplantation had certain similarities in their effects on short-chain fatty acids in feces, and replenished the levels of acetic acid and isobutyric acid. In addition, the approach of microbiota transplantation is closely related to the improvement of disease. The commonly used ways of microbiota transplantation in clinic include upper gastrointestinal tract delivery (esophagogastroduodenoscope, nasogastric tube), lower gastrointestinal tract delivery (colonoscopy, fatal enema), oral capsule delivery and colonial transinoscopic enteral tubing (Lo et al., 2024). We performed microbiota transplantation using the convenient route of gavage. Although oral administration of FMT takes into account the survival of gut bacteria in the gastrointestinal tract, studies have shown that pasteurized *A. muciniphila* can improve IBS as well (Meynier et al., 2024). In addition, *A. muciniphila* can improve cognition and regulate metabolism (Kang et al., 2024). After FMT treatment, *A. muciniphila* is significantly increased, and the symptoms of abdominal pain in patients are improved (Sanders et al., 2019). The above results verify that gut microbiota can improve IBS symptoms, and the supplementation of beneficial bacteria is beneficial to alleviate symptoms. In the future, we will further analyze the composition of the donors, such as their microbial composition and metabolites, as well as the presence of drugs and their active metabolites.

**Figure 7:**
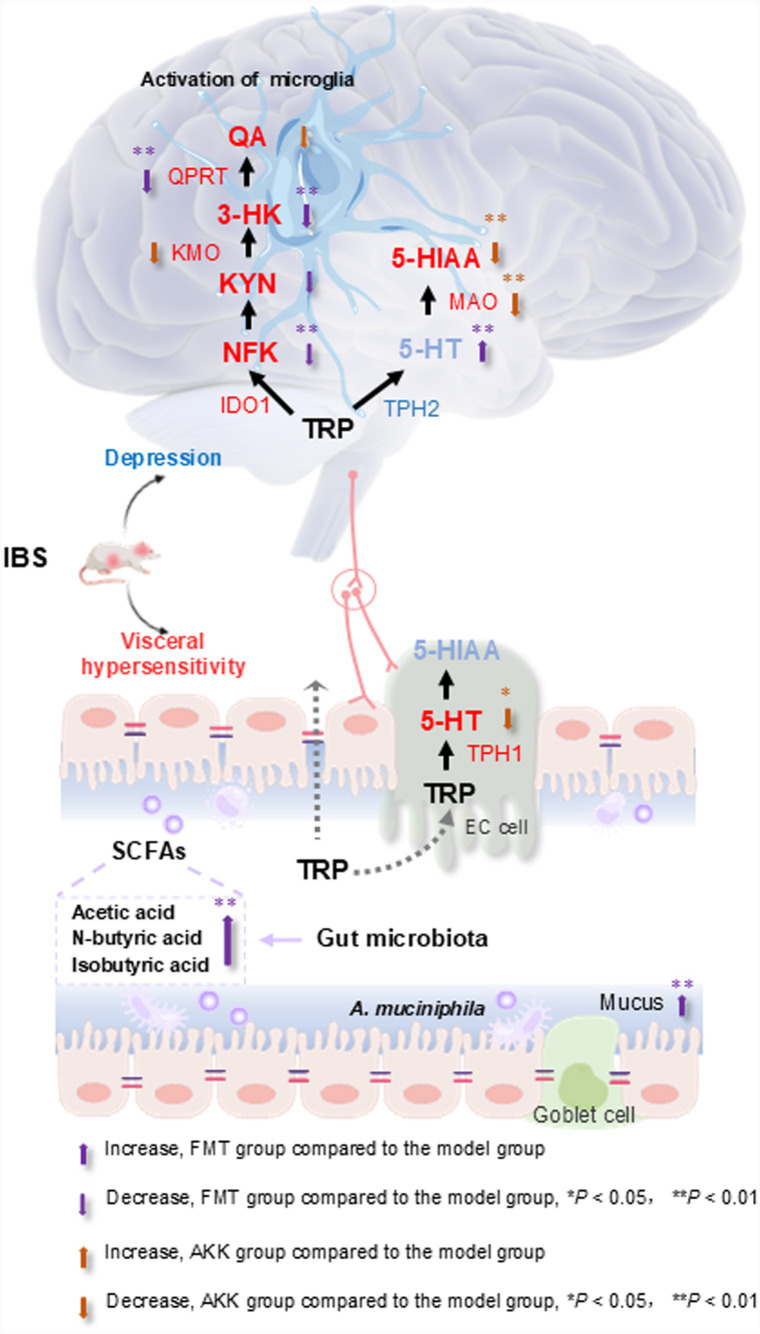
Regulating the microbiota-gut-brain axis is the key for Wuji Pill to improve IBS. Wuji Pill in the treatment of IBS encompass the repair of the intestinal mucus secretion, the modulation of the abnormal activation of microglia, the regulation of the microbial composition and the levels of microbial metabolites, as well as the regulation of gut-brain tryptophan metabolism. TRP, Tryptophan; 5-HT, 5-hydroxytryptamine; 5-HIAA, 5-hydroxyindoleacetic acid; NFK, N-formylkynurenine; KYN, Kynurenine; 3-HK, 3-hydroxykynurenine; QA, Quinolinic acid; IDO, Indoleamine 2,3-dioxygenase; KMO, Kynurenine 3-monooxygenase; QPRT, Quinolinic acid phosphoribosyl Transferase; MAOA, Monoamineoxidase-A; MAOB, Monoamineoxidase-B; TPH, Tryptophan hydroxylase; SCFAs, Short chain fatty acids; IBS, irritable bowel syndrome; *A. muciniphila*, *Akkermansia muciniphila*.

The regulation of tryptophan metabolism between gut microbiota and the host is an important component of the MGB axis (Nunzi et al., 2025). Tryptophan is metabolized mainly through KYN, 5-HT and indole pathways (Xue et al., 2023). Studies have pointed out that the decrease of 5-HT in brain tissue may be one of the causes of depression (Clarke et al., 2013). When the tryptophan-kynurenine pathway is over activated, the content of tryptophan in the brain will be reduced and the synthesis of 5- HT will be reduced (Bender, 1983). In addition, when KMO and QPRT enzymes were over activated, the neurotoxic metabolite quinolinic acid levels were increased (Agus et al., 2018). Quinolinic acid leads to toxicity by preventing the reuptake of glutamate in astrocytes, leading to the activation of microglia and the death of neurons (Garrison et al., 2018). In our results, FMT and *A. muciniphila* transplantation could inhibit the activation of cortical microglia in IBS rats. In the hippocampus of chronic unpredictable mild stress-induced depressed rats, the levels of tryptophan decreased, quinolinic acid increased, and kynurenine/tryptophan increased (Li et al., 2020). The mRNA expressions of IDO1, KMO and QPRT were increased in the hippocampus of IBS rats, which activated the tryptophan-kynurenine pathway. The expression of TPH2 mRNA and 5-HT in hippocampus decreased, while the expression of MAOA and MAOB mRNA increased, and 5-HT was transformed into 5-hydroxyindoleacetic acid. FMT and *A. muciniphila* could inhibit the expression of KMO and QPRT mRNA and reduce the production of quinolinic acid. At the same time, FMT and *A. muciniphila* inhibited the mRNA expression of MAOA and MAOB, 5-HT conversion was reduced. In brief, there is a positive correlation between IBS rats and the TRP-KYN axis. The level of 5-HT in IBS rats is significantly decreased. The levels of 5-HT, 3-HK, and NFK in FMT group are mainly regulated, while after *A. muciniphila* transplantation, the level of QA is mainly reduced. In addition, the level of 5-HT in the colon of IBS rats was increased. The increase of 5-HT in the colon may be one of the reasons for abnormal colon movement and increased visceral sensitivity in patients with IBS. FMT and *A. muciniphila* intervention reduced the level of 5-HT. In summary, the mechanism of Wuji Pill in improving IBS may be related to the regulation of tryptophan metabolism pathways and associated enzymes by gut microbiota. These results were summarized in Figure 7.

## 5 Conclusion

In summary, the present study reported that Wuji Pill can improve intestinal dysfunction and depression-like behavior of IBS rats through MGB axis. Furthermore, Administration of Wuji Pill restores the function of the intestinal mucous barrier, regulates the gut microbiota. It is worth noting that in the experiment of microbiota transplantation, regulating the gut microbiota may be an effective way for Wuji Pill to improve IBS. FMT and *A. muciniphila* can improve the symptoms of IBS rat by increasing intestinal mucus secretion, elevating the levels of SCFAs, and regulating the tryptophan metabolic pathway. Overall, these findings provide new insights for TCM to alleviate IBS complicated with depression and offer strategies for the treatment of diseases related to DGBI.

## Data Availability

The datasets presented in this study can be found in online repositories. The names of the repository/repositories and accession number(s) can be found at: https://ngdc.cncb.ac.cn/gsa, GSA: CRA023334.
