# Supplementary material for: Wuji Pill and Akkermansia muciniphila alleviates intestinal dysfunction and depression-like behavior in irritable bowel syndrome through the microbiota-gut-brain axis
Source: Front Microbiol. 2026 Feb 4;17:1739408. doi: 10.3389/fmicb.2026.1739408 (PMC12913531; doi:10.3389/fmicb.2026.1739408)
Supplement: Supplementary file 1 [file Supplementary_file_1.docx]

Supplementary Material

# Supplementary Table

**Table S1** UPLC-Q-TOF-MS/MS data of the chemical composition of Wuji Pill.

| **No.** | **RT  (min)** | **Chemical composition** | **Formula** | **Theoretical  (m/z)** | **Measured  (m/z)** | **Fragment ion** | **Source** |
| --- | --- | --- | --- | --- | --- | --- | --- |
| 1 | 1.76 | wuchuyuamide Ⅰ | C_19_H_19_N_3_O | 305.1528 | 306.1589[M+H]^+^ | 305.1516, 133.0738 | Fructus Evodiae |
| 2 | 2.22 | paeonilactone C | C_17_H_18_O_6_ | 318.1103 | 319.1160[M+H]^+^ | - | Radix Paeoniae Alba |
| 3 | 2.59 | albiflorin | C_23_H_28_O_11_ | 480.1631 | 525.1555[M+COOH]^－^ | 479.1401 | Radix Paeoniae Alba |
| 4 | 3.41 | coptisine | C_19_H_14_NO_4_ | 320.0922 | 338.1273[M+NH_4_]^+^ | 320.0935, 278.0702, 262.0744, 292.0866, 291.0781 | Rhizoma Coptidis |
| 5 | 3.45 | epiberberine | C_20_H_18_NO_4_ | 336.1235 | 354.1554[M+NH_4_]^+^ | 320.1217, 321.1289, 307.1113, 292.121 0 | Rhizoma Coptidis |
| 6 | 3.46 | berberine | C_20_H_18_NO_4_ | 335.1157 | 353.1520[M+NH_4_]^+^ | 292.1210, 307.1113, 308.1191, 320.1217, 335.1182 | Rhizoma Coptidis |
| 7 | 3.51 | *β*-sitosterol | C_29_H_50_O | 414.3861 | 453.3459[M+K]^+^ | 209.1506, 304.6636, 435.3339 | Rhizoma Coptidis |
| 8 | 3.85 | palbinone | C_22_H_30_O_4_ | 358.2144 | 359.2244[M+H]^+^ | 358.2171 | Radix Paeoniae Alba |
| 9 | 4.15 | 12*α*-hydroxylimonin | C_26_H_30_O_8_ | 486.1889 | 487.1965[M+H]^+^ | 441.1849, 469.1860, 336.1123 | Fructus Evodiae |
| 10 | 5.33 | 2',4,4'-trihydroxy-6'-methoxy-dihydrochalcone | C_16_H_14_O_5_ | 288.0997 | 306.1346[M+NH_4_]^+^ | 306.1346, 274.0800, 106.0435 | Rhizoma Coptidis |
| 11 | 5.34 | 7*β*-hydroxyrutaecarpine | C_18_H_13_N_3_O_2_ | 303.1007 | 302.0940[M－H]^－^ | 303.0976, 304.0992, 302.0945, 142.0192, 167.8269 | Fructus Evodiae |
| 12 | 5.59 | cyclo-(phe-leu) | C_15_H_20_N_2_O_2_ | 260.1524 | 283.1429[M+Na]^+^ | 283.143 | Rhizoma Coptidis |
| 13 | 5.86 | paeoniflorigenone | C_17_H_18_O_6_ | 318.1103 | 319.1174[M+H]^+^ | 317.2625,181.0796 | Radix Paeoniae Alba |
| 14 | 5.95 | *N*-trans-feruloyltyramine | C_18_H_19_NO_4_ | 313.1314 | 331.1639[M+NH_4_]^+^ | 313.1301 | Rhizoma Coptidis |
| 15 | 6.07 | oxysanguinarine | C_20_H_13_NO_5_ | 347.0793 | 348.3162[M+H]^+^ | 290.0732, 347.3131 | Rhizoma Coptidis |
| 16 | 6.1 | *Z*-octadecyl caffeate | C_27_H_44_O_4_ | 432.3239 | 431.3169[M－H]^－^ | 432.3242 | Rhizoma Coptidis |
| 17 | 6.2 | sanguinarine | C_20_H_14_NO_4_ | 332.0922 | 350.1235[M+NH_4_]^+^ | 317.0943, 216.9777, 304.1289, 302.1277, 289.1335 | Rhizoma Coptidis |
| 18 | 6.82 | dihydrodehydrodiconiferyl alcohol | C_20_H_24_O_6_ | 360.1572 | 378.1938[M+NH_4_]^+^ | 186.0659, 360.1600 | Rhizoma Coptidis |
| 19 | 7.2 | lactiflorin | C_23_H_26_O_10_ | 462.1525 | 480.1912[M+NH_4_]^+^ | 463.1361, 462.1574, 301.1003, 167.5052 | Radix Paeoniae Alba |
| 20 | 7.23 | 11*α*,12*α*-epoxy-23*β*,23-dihydroxyolean-28,13*β*-olide | C_30_H_46_O_6_ | 502.3294 | 503.3332[M+H]^+^ | 502.3289 | Radix Paeoniae Alba |
| 21 | 7.45 | paeonilactone B | C_10_H_12_O_4_ | 196.0735 | 197.0799[M+H]^+^ | 196.0726, 179.0538, 161.0145 | Radix Paeoniae Alba |
| 22 | 7.46 | (+)-5'-methoxylariciresinol | C_22_H_28_O_8_ | 420.1784 | 421.1840[M+H]^+^ | 119.9302, 120.9893, 147.8989, 205.0839, 391.1846, 420.1767 | Rhizoma Coptidis |
| 23 | 7.58 | magnoflorine | C_20_H_24_NO_4_ | 342.1705 | 360.2035[M+NH_4_]^+^ | 265.1350, 282.0249, 297.0757, 299.0747, 342.1697 | Rhizoma Coptidis |
| 24 | 7.67 | *P*-hydroxyphenethyl trans-ferulate | C_18_H_18_O_5_ | 314.1154 | 332.1461[M+NH_4_]^+^ | 314.1123, 207.0682, 195.0684, 133.031 9 | Rhizoma Coptidis |
| 25 | 7.85 | *N*-（2-methylaminobenzoyl）tryptamine | C_18_H_19_N_3_O | 293.1528 | 311.1881[M+NH_4_]^+^ | 133.0300, 29 3.1773, 294.1813 | Fructus Evodiae |
| 26 | 8.14 | hydroxyevodiamine | C_19_H_17_N_3_O_2_ | 319.132 | 337.1657[M+NH_4_]^+^ | 187.0779, 319.1929, 320.196 5 | Fructus Evodiae |
| 27 | 8.15 | kaempferol 3,7-di-*O*-glucoside | C_27_H_30_O_16_ | 610.1533 | 633.1451[M+Na]^+^ | 611.5664, 610.1559 | Radix Paeoniae Alba |
| 28 | 8.26 | demethyleneberberine | C_19_H_18_NO_4_ | 324.1235 | 363.0884[M+K]^+^ | 309.1845 | Rhizoma Coptidis |
| 29 | 8.74 | daucosterol | C_35_H_60_O_6_ | 576.4389 | 577.4457[M+H]^+^ | 221.1348, 133.0294, 615.4403, 576.4384 | Radix Paeoniae Alba |
| 30 | 8.75 | 6-*O*-*β*-D-glucopyranosyllactinolide | C_16_H_26_O_9_ | 362.1576 | 380.1888[M+NH_4_]^+^ | 363.1508,362.1255 | Radix Paeoniae Alba |
| 31 | 8.8 | worenine | C_20_H_16_NO_4_ | 334.1079 | 335.1129[M+H]^+^ | 261.1120, 302.1407, 304.9031, 334.105 7 | Rhizoma Coptidis |
| 32 | 8.97 | (+)-pinoresinol glucoside | C_26_H_32_O_11_ | 520.1944 | 543.1993[M+Na]^+^ | 519.1738, 387.1113, 369.1887 | Rhizoma Coptidis |
| 33 | 9.61 | 11-methoxy-5-methyl-1,3-dioxolo[4,5-b]acridin-10(5*H*)-one | C_16_H_13_NO_4_ | 283.0844 | 301.1187[M+NH_4_]^+^ | —— | Fructus Evodiae |
| 34 | 9.62 | 2,3-bis[(4-hydroxy-3,5-dimethoxyphenyl)-methyl]-1,4-butanediol | C_20_H_26_O_6_ | 362.1729 | 380.2065[M+NH_4_]^+^ | 380.2065 | Rhizoma Coptidis |
| 35 | 9.65 | 12-*α*-hydroxyevodol | C_26_H_28_O_10_ | 500.1682 | 499.1605[M－H]^－^ | 439.1306, 500.1650, 501.1887 | Fructus Evodiae |
| 36 | 9.87 | longifolroside A | C_27_H_34_O_11_ | 534.2101 | 552.2451[M+NH_4_]^+^ | 301.1361 | Rhizoma Coptidis |
| 37 | 9.97 | *N*，*N*-dimethyl-5-methoxytryptamine | C_13_H_18_N_2_O | 218.1419 | 219.1495[M+H]^+^ | 175.0850, 218.1422 | Fructus Evodiae |
| 38 | 10.54 | 14-formyldihydrorutaecarpine | C_19_H_15_N_3_O_2_ | 317.1164 | 335.1521[M+NH_4_]^+^ | 144.8829, 289.1256, 290.1259, 316.9405, 338.2033, 317.1183 | Fructus Evodiae |
| 39 | 10.75 | evodiamine | C_19_H_17_N_3_O | 303.1371 | 304.1430[M+H]^+^ | 303.1428, 275.1089, 165.9600 | Fructus Evodiae |

In prior investigations, chemical constituent identification of the Wuji Pill extract was conducted utilizing UPLC-Q-TOF-MS/MS(Guo, Yuxuan et al., 2022).

Abbreviations: RT, Retention Time.

# Supplementary Figures


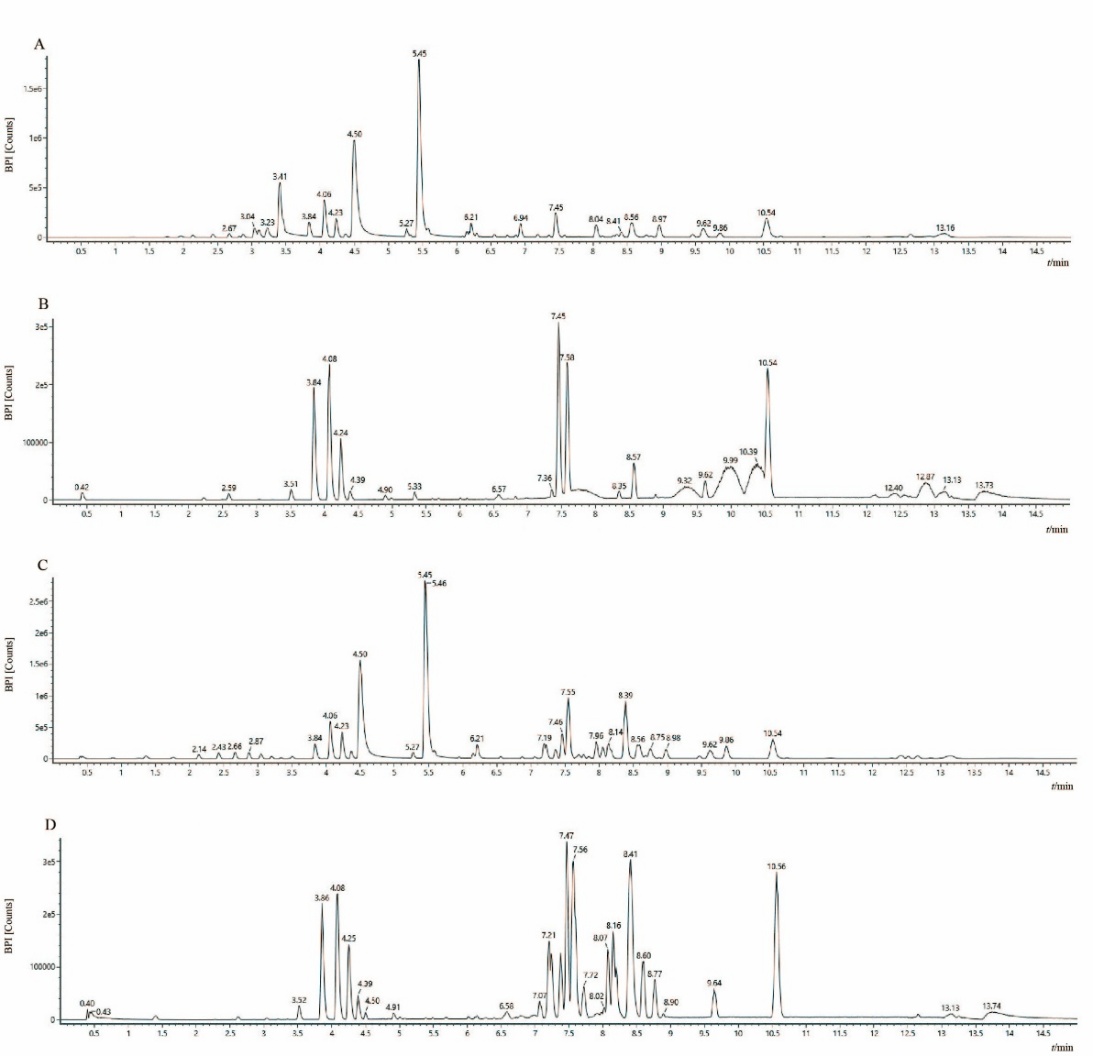


**Fig. S1** Total ion chromatograms in positive and negative ion modes. In previous studies, qualitative chemical profiling of Wuji Pill was conducted utilizing UPLC-Q-TOF-MS/MS. (A) Wuji Pill positive ion modes; (B) Wuji Pill negative ion modes; (C) Wuji Pill -containing plasma sample positive ion modes; (D) Wuji Pill -containing plasma sample negative ion modes (Guo, Yuxuan et al., 2022).

**
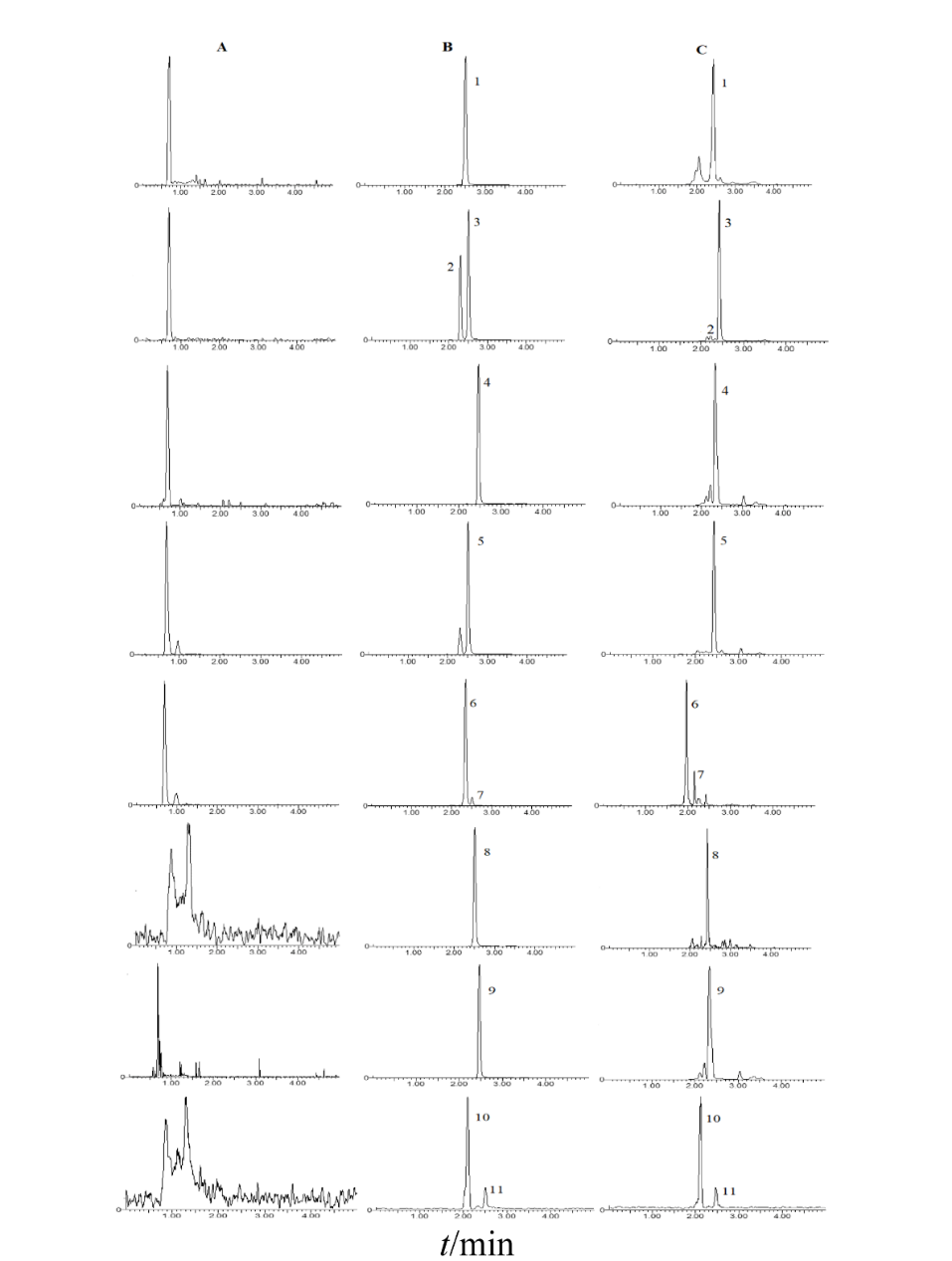
**

**Fig. S2** The UPLC-MS/MS chromatogram of 10 active components of Wuji Pill and the internal standard diphenhydramine in rat plasma. In previous studies, quantitative detection of 10 active components in Wuji Pill was performed using UPLC-MS/MS. (A) Blank plasma; (B) Blank plasma and reference substance; (C) Plasma sample after intragastric administration of Wuji Pill for 5 min; (1) Diphenhydramine (internal standard); (2) Berberine; (3) Epiberberine; (4) Palmatine; (5) Coptisine; (6) Jatrorrhizine; (7) Dihydroberberine; (8) Evodiamine; (9) Evodin; (10) Paeoniflorin; (11) Albiflorin.

**
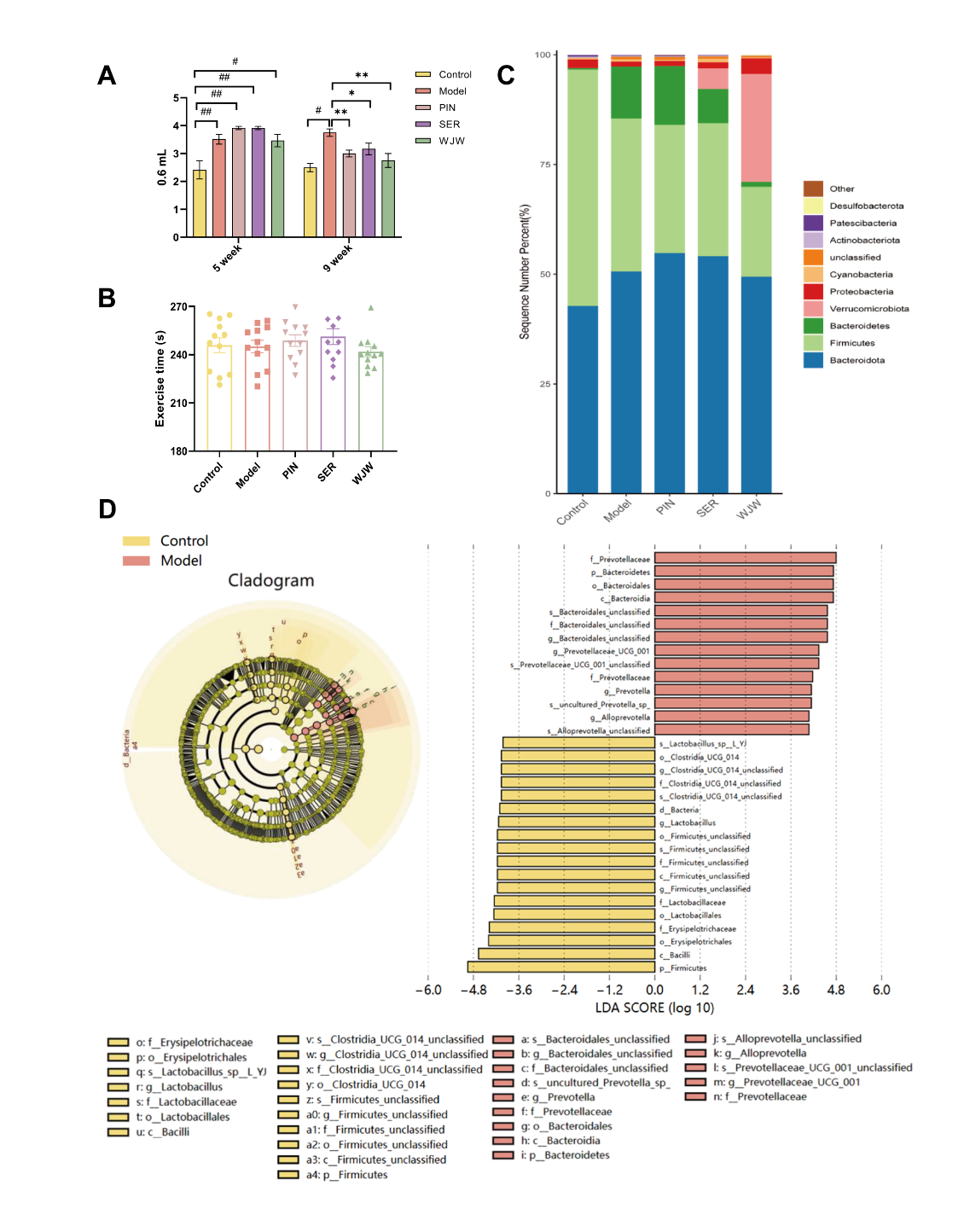
**

**Fig. S3** Wuji Pill ameliorates IBS symptoms and regulates gut microbiota. (a) The results of the AWR score (n = 12). (b) Open field test (n = 12). (c) Analysis of the gut microbiota taxon composition. (d) Gut microbiota LEfSe analysis. Data are presented as the mean ± SEM, compared with the control group, *#P* < 0.05, *##P* < 0.01; compared with the model group, **P* < 0.05, ***P* < 0.01.

**
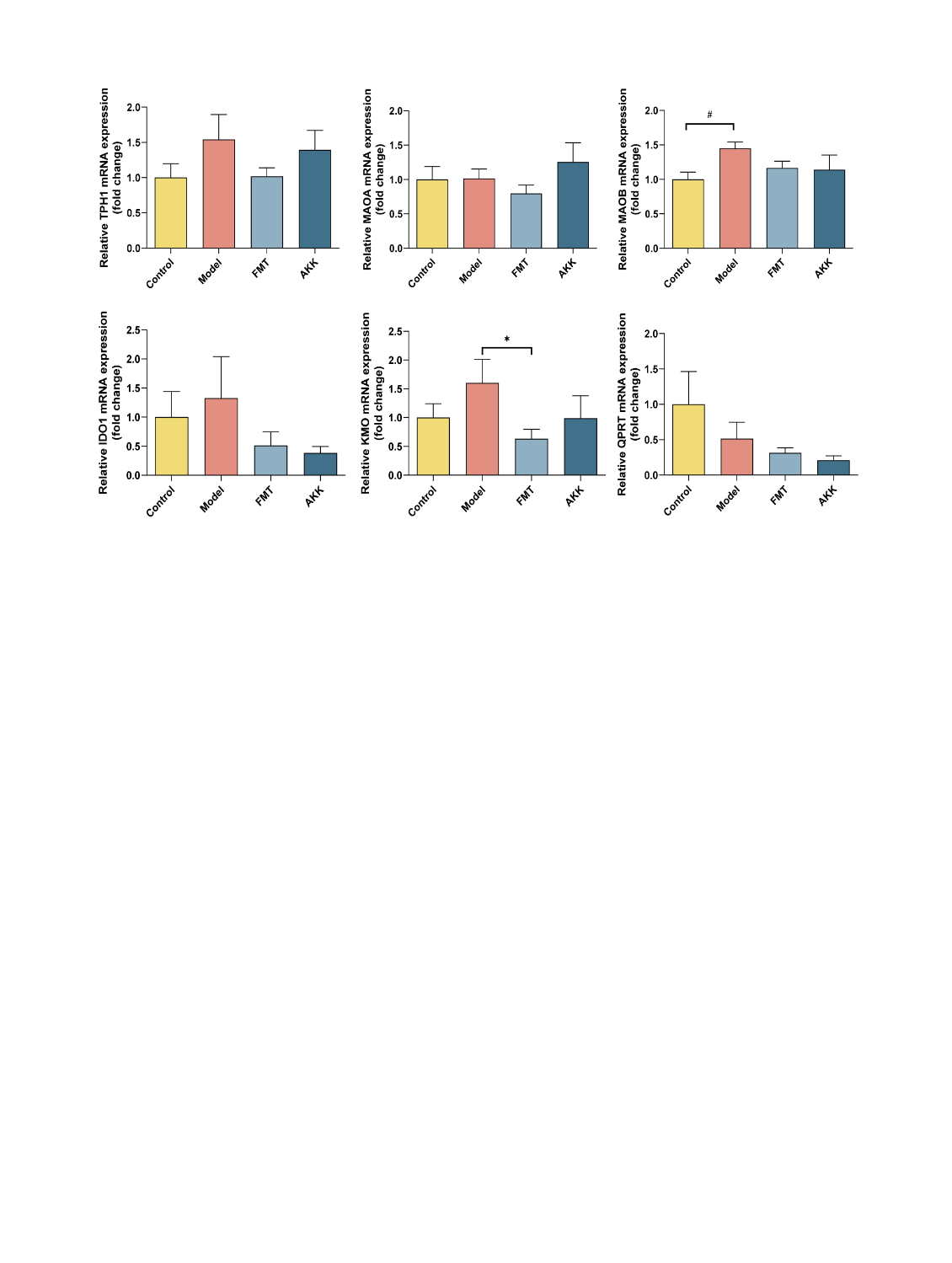
**

**Fig. S4** Microbiota transplantation affects the enzymes in the tryptophan metabolism pathways. The expression of tryptophan metabolic enzymes in colon (n = 6). Data are presented as the mean ± SEM, compared with the control group, *#P* < 0.05; compared with the model group, **P* < 0.05.

# References

Guo, Yuxuan, Zhang, Shuhan, Wang Anqi, Zhu, Xiaoxin, Li, Yujie, Chen, Ying, et al. (2022). Pharmacodynamic substances and therapeutic potential of wuji pills: Based on UPLC-Q-TOF-MS/MS and network pharmacology. *China journal of Chinese materia medica* 47, 6720–6729. doi: 10.19540/j.cnki.cjcmm.20220727.702
